# Prediction of Knee Joint Compartmental Loading Maxima Utilizing Simple Subject Characteristics and Neural Networks

**DOI:** 10.1007/s10439-023-03278-y

**Published:** 2023-06-19

**Authors:** Jere Lavikainen, Lauri Stenroth, Tine Alkjær, Pasi A. Karjalainen, Rami K. Korhonen, Mika E. Mononen

**Affiliations:** 1 Department of Technical Physics, University of Eastern Finland, Kuopio, Finland; 2 Department of Biomedical Sciences, University of Copenhagen, Copenhagen, Denmark; 3 Parker Institute, Bispebjerg and Frederiksberg Hospital, Copenhagen, Denmark

**Keywords:** Knee joint, Contact force, Compartmental loading, Knee osteoarthritis, Gait analysis, Artificial neural networks, OpenSim

## Abstract

Joint loading may affect the development of osteoarthritis, but patient-specific load estimation requires cumbersome motion laboratory equipment. This reliance could be eliminated using artificial neural networks (ANNs) to predict loading from simple input predictors. We used subject-specific musculoskeletal simulations to estimate knee joint contact forces for 290 subjects during over 5000 stance phases of walking and then extracted compartmental and total joint loading maxima from the first and second peaks of the stance phase. We then trained ANN models to predict the loading maxima from predictors that can be measured without motion laboratory equipment (subject mass, height, age, gender, knee abduction-adduction angle, and walking speed). When compared to the target data, our trained models had NRMSEs (RMSEs normalized to the mean of the response variable) between 0.14 and 0.42 and Pearson correlation coefficients between 0.42 and 0.84. The loading maxima were predicted most accurately using the models trained with all predictors. We demonstrated that prediction of knee joint loading maxima may be possible without laboratory-measured motion capture data. This is a promising step in facilitating knee joint loading predictions in simple environments, such as a physician’s appointment. In future, the rapid measurement and analysis setup could be utilized to guide patients in rehabilitation to slow development of joint disorders, such as osteoarthritis.

**Supplementary Information:**

The online version contains supplementary material available at 10.1007/s10439-023-03278-y.

## Introduction

Walking and other forms of human locomotion put joints, particularly the knee joint, under stress. Excess loading of the knee joint may have adverse effects on the health and pathology of the joint [13]. For instance, loading of the knee joint may affect the development of knee osteoarthritis (KOA), a disease that affects both the tibiofemoral and the patellofemoral knee joints, causes pain and immobilization, and has major healthcare costs worldwide [18, 32]. Although a causal link between knee joint loading (estimated by knee adduction moments and derived measures as well as tibiofemoral compression force) and structural disease progression of KOA cannot be plausibly established [17], knee joint loading is believed to have an important role in the development and progression of KOA particularly in the context of frontal plane malalignment and excess loading in either compartment of the joint [9, 13]. Personalized knee joint loading distributions can be estimated by knee joint contact forces, which in turn can be solved with musculoskeletal (MS) modeling utilizing various motion capture (MOCAP) setups [10]. Because information about knee joint loading can be utilized to instruct better movement strategies, capabilities of providing personalized estimations for the joint loading distributions could enable teaching patients movements that reduce loading on vulnerable parts of the joint. However, the biggest limitation why this is not possible in clinics is due to availability of MOCAP setups. This indicates unmet needs for simpler solutions to be applied in the clinics.

MS modeling requires measuring experimental MOCAP data and several computational analysis steps to retrieve knee joint contact forces (KJCFs). Experimentally measured data includes trajectories of markers placed on the subject’s skin and ground reaction forces (GRFs) measured with force plates; it may also include other data, such as electromyographic (EMG) signals [2] or data from inertial measurement units [30]. The placing of the markers requires experience and time. The measurement technologies are generally not portable and thus, necessitate measurements in a motion laboratory. Additionally, the analysis steps required to calculate KJCFs are time-consuming. This cumbersomeness may be acceptable in a research setting but can exclude the calculation of KJCFs from clinical use where patient comfortability and time are limiting factors. Therefore, less cumbersome methods to retrieve KJCFs are required. Machine learning methods such as artificial neural networks (ANNs) present a potential solution.

Predicting KJCFs with effortlessly measurable input data could make the estimation of knee joint loading portable. While some previous studies have used raw MOCAP data such as marker trajectories and GRF signals as the input to predict KJCFs in the medial compartment of the knee joint using ANNs [2, 28], the use of predictors with more intuitive biomechanical connection to knee joint loading could be justified and simplify the experimental measurements. For example, the subject’s mass, height, BMI, and walking speed have been used as predictors to predict medial KJCF peaks using ANNs [4, 20]. However, those studies still included joint moments [4] or joint angles [12, 20] among the predictors, meaning that MOCAP data were still required in the prediction.

Clearly, the inconvenience of MOCAP data has been noticed by biomechanics researchers and existing studies show a trend toward dynamics estimation while skipping the time-consuming MS analysis steps [2, 12, 20, 28] and using light measurement setups (e.g., without full marker data) instead of laboratory-grade MOCAP [20, 30]. Eliminating reliance on MOCAP-based input data would be a significant step forward in the prediction of the peaks or entire time series of compartmental KJCFs. If KJCFs could be predicted with sufficient accuracy using demographic and anthropometric data, the biomechanical joint loading-based risk assessment of KOA or similar conditions could be done much faster than is possible when MOCAP is required. However, it should be noted that this would require very large training data sets, which would have to consider all possible variations in subject characteristics (such as age, height, weight, type of knee injury).

In this study, we trained feedforward ANNs to predict the total and compartmental KJCFs peaks using mostly anthropometric and demographic data as the input. The input data comprised subject mass, height, age, gender, knee abduction-adduction angle during static standing, and walking speed. Using the same input data, we also investigated the accuracy of predicting medial force ratios (MFRs), which describe the compartmental distribution of joint loading. We aimed to answer the following research questions:

- How accurately can medial, lateral, and total KJCF loading response, terminal extension, and full-stance peaks be predicted using ANNs without motion capture data?
- How accurately can loading response, terminal extension, and full-stance MFRs be predicted using ANNs without motion capture data?
- What is the inter-trial variability of KJCF peaks, i.e., what is the theoretical upper limit of prediction accuracy?

## Materials and Methods

**Fig. 1:**
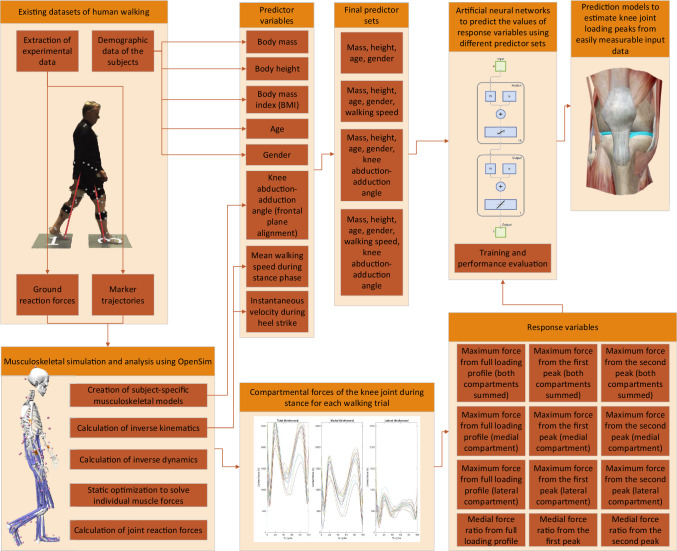
The workflow consists of extracting data from existing datasets, conducting musculoskeletal simulation and analysis, and constructing artificial neural networks. The data from existing datasets include demographic data and motion capture data. Demographic data are used to formulate most of the predictor variables, while motion capture data are analyzed to retrieve the remaining predictor variables and all response variables. Finally, artificial neural networks are trained for each combination of predictor sets and response variables to predict the value of the response variable from the predictor variables in the predictor set. Detailed information about training the artificial neural network can be found in the Supplementary material

The study workflow is presented in Fig. 1.

### Included Datasets

The combined dataset for the study was constructed from five separate original datasets. All original datasets contained information about the height, weight, gender, and age of the participants in addition to lower body marker trajectories and ground reaction forces (GRFs) during level walking.

The first original dataset was the CAROT [1] dataset, which contained participants of both genders who had been diagnosed with osteoarthritis in at least one knee. The pathological leg was used in the analysis. Most participants in the dataset had trials from several measurement sessions, in which case we utilized all viable sessions. The CAROT study was approved by the Scientific Ethical Committees for the Capital Region, Denmark (H-B-2007-088). Additionally, we used data from four open datasets: datasets by Schreiber and Moissenet [29]; Fukuchi et al. [11]; Horst et al. [19]; and Camargo et al. [5]. They comprised healthy participants of both genders and different ages. All open datasets reported informed consent from the participants and ethics approval from an appropriate body.

The combined dataset consisted of 124, 49, 42, 54, and 21 subjects from the CAROT, Schreiber, Fukuchi, Horst, and Camargo datasets, respectively. The combined dataset covers most but not all of the subjects in the original datasets because some subjects had too little valid data (elaborated below in “Estimation Of Joint Loading” section) to be included. Table 1 shows demographic information about the combined dataset and the individual datasets that constitute it. Visual illustrations of the response variables and their dispersion are presented in the Supplementary material.

**Table 1:** Demographic information about the subjects in the combined dataset, presented per original dataset and in the combined dataset as a whole

Dataset	Age (years)			Mass (kg)			Height (m)			BMI (kg/m^2^)			Gender	
	Min	Median	Max	Min	Median	Max	Min	Median	Max	Min	Median	Max	m	f
Camargo	19	21	33	52.2	68.0	96.2	1.52	1.73	1.80	18.6	23.6	30.3	13	8
CAROT	50	62	78	74.0	99.3	145	1.49	1.65	1.91	28.2	35.9	51.0	25	99
Fukuchi	21	33	84	44.9	68.5	95.4	1.47	1.68	1.92	18.0	24.0	33.1	24	18
Horst	19	22	30	47.3	69.0	94.2	1.55	1.77	1.99	18.5	22.2	26.7	28	26
Schreiber	19	38	67	50.0	68.0	98.0	1.55	1.73	1.92	17.2	23.5	29.6	26	23
Combined	19	53	84	44.9	80.8	145	1.47	1.69	1.99	17.2	26.7	51.0	116	174

The presented mass and BMI values for CAROT dataset, where there were several measurement sessions and intra-subject mass and BMI changed between them, represent the values from the first measurement session

### Estimation of Joint Loading

We used the open-source musculoskeletal (MS) simulation and analysis software, OpenSim [7], to estimate knee joint contact forces (KJCF) using the experimental marker trajectories and ground reaction forces (GRF) of the datasets (Fig. 1). The analysis pipeline included the following steps: experimental data extraction, creation of subject-specific musculoskeletal model by scaling the generic model, calculation of inverse kinematics (IK) and inverse dynamics (ID), static optimization (SO) to solve individual muscle forces, and finally the calculation of joint reaction forces. We implemented this pipeline as MATLAB scripts that invoked the OpenSim 4.1 application programming interface. Details of musculoskeletal modeling and simulation, including validation of the selected analysis pipeline, can be found in the Supplementary material.

Before constructing the combined dataset, the estimated KJCF curves were visually checked to exclude trials with potential measurement, modeling, and simulation-related error to ensure validity of the data entering further analyses. Trials were excluded based on non-physiological reasons, such as simulation-related artifacts or technical issues. Exclusion was done on a per-trial basis rather than on a per-subject basis, i.e., presence of excluded trials did not prevent the inclusion of valid trials under the same subject in the combined dataset. Common criteria for exclusion were the presence of only half of the stance phase and sharp distortions that would have been detected as peak values in the following analysis step. Reasons for them included the stance phase-detecting algorithm mistaking the mid-stance local minimum as the end-of-stance minimum in force plate data, jumping marker labels that caused sudden changes in IK, and distortions in force plate data. Overall, 19% of analyzed trials were excluded based on visual inspection of KJCF curves. Although some of these errors could have been corrected by trial-specific manual changes in the data and analysis pipeline, those trials were excluded instead of corrected because fixing them would have taken a lot of time and because of the large amount of valid data we received with the automatic analysis pipeline. Therefore, only trials with visually validated KJCF curves constituted the combined dataset. Extraction of KJCF peak values, calculation of medial force ratios, and dataset-specific MS modeling notes are presented in the Supplementary material.

### Predictor Selection

Predictors (i.e., input variables for the prediction model) in the combined dataset were the mass, height, BMI, age, gender, knee angle, walking speed, and heel strike velocity of the subject (Fig. 1). They were selected due to availability and the possibility of simple acquisition, e.g., in a clinical setting. Knee angle was defined as the abduction-adduction angle of the knee during static standing where positive values indicated adduction and negative values indicated abduction. It was determined for each subject during the scaling of the musculoskeletal model. The walking speed was calculated as the mean velocity of MOCAP markers on the pelvis along the walking direction during the stance phase. Similarly, heel strike velocity was the momentary velocity of the pelvis in three-dimensional space between the frame in the marker data where heel strike was detected and the immediately following frame.

To assess predictor collinearity, we calculated variance inflation factors (VIFs) [14] for the predictors. VIF describes the multicollinearity of predictors in a multiple regression model and is a measure of how much explained variance a predictor shares with other predictors. Mass, height, and BMI had VIFs above 20, with walking speed and heel strike velocity both having VIFs above 3. When BMI and heel strike velocity were omitted from the predictor set, the remaining variables had VIFs below 3. This observation confirmed that having BMI, mass, and height or walking speed and heel strike velocity in the predictor set resulted in high collinearity. Therefore, we removed BMI and heel strike velocity from the predictor set; we chose the removal of heel strike velocity over walking speed because walking speed is easier to measure. Based on the simplicity of measuring the predictors, we formed four predictor sets *P*_*A*_, *P*_*B*_, *P*_*C*_, and *P*_*D*_ with varying number of predictors (Table 2).

**Table 2:** Chosen predictor sets and the variables they included

Predictor set	Included predictors
*P*_*A*_	Mass, height, age, gender
*P*_*B*_	Mass, height, age, gender, walking speed
*P*_*C*_	Mass, height, age, gender, knee angle
*P*_*D*_	Mass, height, age, gender, walking speed, knee angle

Measurement simplicity decreases gradually from *P*_*A*_ to *P*_*D*_; *P*_*A*_ comprises only demographic and anthropometric parameters, while *P*_*D*_ contains predictors that are possible to measure outside the motion laboratory with a goniometer, measuring tape, and a stopwatch

### Artificial Neural Network Analysis

MATLAB R2020b (MathWorks, Natick, MA) with deep learning toolbox for the ANN analysis was utilized (Fig. 1). For the architecture of the neural network, we chose a fully connected feedforward network. The ANNs consisted of an input layer, a single hidden layer, and an output layer. In the hidden layer, the activation function, the number of nodes, and the training algorithm were chosen with a hyperparameter optimization algorithm that is described in Supplementary materials. The activation function in the output layer was a linear function.

We used backpropagation training to train ANNs to predict loading response, terminal extension, and full-stance peak on the medial and lateral compartments and the aforementioned peaks identified from the total sum of the time series of both compartments with different predictor sets. Additionally, we trained ANNs to predict MFRs during loading response, terminal extension, and the entire stance phase. Individual trials, and not their ensemble averages, were used in training the ANNs; information about weighting the trials can be found in the Supplementary material. In summary, an ANN was trained for each predictor-output combination, totaling 48 ANNs (four predictor sets times twelve response variables).

During training, all data points in the combined dataset were distributed into three subsets: a training set, a validation set, and a test set. Recommended distributions of data in the sets vary per source but at least half of the whole dataset is usually allocated to the training set [15, 22]; we used fivefold cross-validation to allocate the subsets, explained in detail in the Supplementary material. The data in the training set were used to iteratively modify the weights between the input layer and the hidden layer and the hidden layer and the output layer. MATLAB’s predefined training algorithm was used to update the weights iteratively. Formulation of the weights of the training samples is explained in the Supplementary material.

Finally, according to the cross-validation scheme described in the Supplementary material, we determined test error by evaluating the independent test set in the ANN and calculating the RMSE by comparing the predicted output to the true (i.e., MS model estimated) output. To ensure comparability of ANN prediction accuracy between different predictor sets, we used the same random seed of one to initialize the Mersenne Twister pseudorandom number generator of MATLAB for subset division and initial network weight generation for each predictor set.

### Analysis of Response Variability

Because of inherent variability in repeated movements, knee loading profiles always differ between trials within a single subject. Therefore, no matter how well-trained and well-structured ANNs are used, response variables in an independent dataset are impossible to predict with perfect accuracy. To determine what the highest attainable accuracy is, within each subject and trial configuration, the inter-trial variability of the response variables was evaluated. For each response variable, we grouped the response values under their respective subjects and, if applicable, further under different trial configurations. The trial configurations included instructed walking speed, measurement session, and other parameters so that each group would ideally result in the same knee loading if there was no measurement noise and the subject could replicate their movement exactly. For each set of responses in a group excluding groups with less than four trials, we calculated the standard deviation. We then calculated the mean and standard deviation of the intra-group standard deviations for each original dataset.

We chose standard deviation as the measure of dispersion (i.e., variability) over other options such as range, interquartile range, and mean absolute deviation because standard deviation is more intuitive to compare against RMSE due to the similarities in their equations. RMSE is calculated as

$${\text{RMSE}} = \sqrt {\frac{{\mathop \sum \nolimits_{i = 1}^{N} \left( {y_{i} {-}t_{i} } \right)^{2} }}{N}},$$

where *y*_*i*_ is the *i*-th predicted response, *t*_*i*_ is the *i*-th target value of the response, and *N* is the number of data points. Standard deviation is calculated as

$${\text{STD}} = \sqrt {\frac{{\mathop \sum \nolimits_{i = 1}^{M} \left( {t_{i} - \mu } \right)^{2} }}{M - 1}},$$

where *t*_*i*_ is the *i*-th true value (i.e., target value) of the response, *μ* is the sample mean of all *t*_*i*_, and *M* is the number of *t*_*i*_*s*. If we assume that *y*_*i*_ equals *μ* for all *i* and the number of data points *N* in the test subset that is used to calculate RMSE equals *M* − 1, RMSE and standard deviation are equal. This assumption fails to hold, but we acknowledged this connection between these two measures and treated standard deviation as an approximation of the minimum attainable RMSE for the ANN.

With standard deviation, we had approximate information of how much the response values vary when measurement conditions remain unchanged. This information provided the context necessary for interpreting the prediction accuracy of the ANNs.

## Results

The KJCF peaks resulting from the musculoskeletal (MS) analysis pipeline are available online on Zenodo (https://zenodo.org/record/7253458) [23]. The online dataset also contains the predictors corresponding to the KJCF peaks so that others may use the data in their prediction algorithms. The dataset contains data only from the four open datasets.

### Analysis of Response Variability

In Table 3, for all 12 response variables, we present the means and standard deviations of the standard deviations of KJCF peaks over similar trial conditions with four or more trials a group (i.e., measures that describe how much MS modeling estimated loading peaks vary between trials that are identical in terms of subject characteristics and instructed walking speed). The results vary per original dataset and response variable, but overall the standard deviations of the measures are in the same order of magnitude as the measures themselves.

**Table 3:** Mean and standard deviation (STD) of the STDs of musculoskeletal model estimated KJCF peaks of all similar trial conditions with at least four trials, presented for each response variable and original dataset

Mean ± STD	Camargo	CAROT	Fukuchi	Horst	Schreiber
Full (summed)	378.5 ± 296.0	216.0 ± 149.6	256.4 ± 153.6	270.1 ± 111.4	171.8 ± 147.6
Full (medial)	271.1 ± 192.3	163.5 ± 94.61	208.5 ± 133.4	213.6 ± 82.5	126.4 ± 95.06
Full (lateral)	272.9 ± 185.7	120.7 ± 91.77	130.6 ± 87.61	90.47 ± 55.66	92.65 ± 65.14
LR (summed)	420.2 ± 272.0	299.1 ± 213.9	317.0 ± 193.7	199.6 ± 112.6	213.2 ± 203.3
LR (medial)	307.2 ± 176.5	200.5 ± 139.4	217.4 ± 139.9	118.2 ± 75.63	144.6 ± 125.7
LR (lateral)	282.4 ± 187.3	131.5 ± 92.55	170.4 ± 88.40	105.3 ± 57.79	131.9 ± 65.85
TE (summed)	340.1 ± 273.5	195.2 ± 124.3	238.9 ± 149.5	280.5 ± 84.39	139.4 ± 90.16
TE (medial)	260.2 ± 205.6	162.3 ± 93.24	212.2 ± 133.5	225.5 ± 69.11	120.1 ± 68.0
TE (lateral)	204.5 ± 120.2	93.48 ± 62.21	100.0 ± 71.2	76.73 ± 25.20	81.27 ± 58.07

“Full” refers to the peaks from the full stance, while LR and TE refer to the loading response (first peak) and terminal extension (second peak) phases of stance, respectively. “Summed” refers to the sum of medial and lateral compartment loading. All values are in newtons

### Prediction Accuracy of the Artificial Neural Networks

The best Pearson correlation coefficient (R = 0.84) and root mean square error normalized to the mean of the response variable (NRMSE = 0.14) were reached for the summed peak of both compartments over the full-stance phase with predictor set *P*_*D*_ (Table 4). With predictor sets *P*_*B*_ and *P*_*D*_, the most accurately predicted response variable was the summed peak of both compartments over the full-stance phase (*R* = 0.82 for *P*_*B*_ and *R* = 0.84 for *P*_*D*_), whereas with predictors sets *P*_*A*_ and *P*_*C*_, the summed terminal extension peak was predicted the most accurately (R = 0.74 for P_A_ and *R* = 0.74 for *P*_*C*_).

**Table 4:**
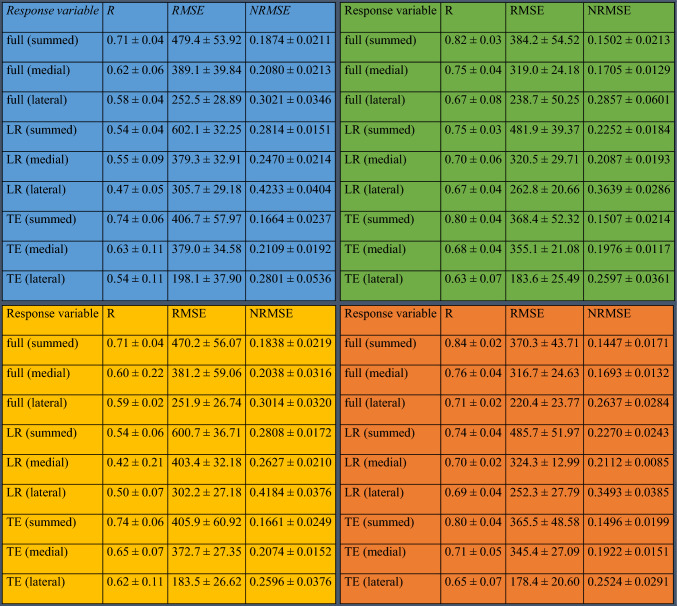
Pearson correlation coefficients (R), root mean square errors (RMSE) between the MS model estimated “ground truth” and artificial neural network-predicted response values, and RMSE normalized to the mean of the response variable (NRMSE) ± the standard deviations of R, RMSE, and NRMSE

RMSE is in newtons. The results are calculated with predictor set P_A_ (mass, height, age, gender), P_B_ (mass, height, age, gender, walking speed), P_C_ (mass, height, age, gender, knee angle), and P_D_ (mass, height, age, gender, walking speed, knee angle) in the top left corner (blue), top right corner (green), bottom left corner (orange), and bottom right corner (red), respectively

The worst attained correlation between predicted and MS model estimated responses was for the medial loading response peak with predictor set *P*_*C*_ (*R* = 0.42), and the worst NRMSE was for the lateral loading response peak with predictor set P_A_ (NRMSE = 0.42). In both predictor sets, the loading response peaks always performed worse than terminal extension peaks or full-stance peaks.

When walking speed was included in the predictor set, prediction accuracy improved without exception compared with a similar predictor set without walking speed. While compartmental KJCF peaks can be predicted with *R* > 0.4 with just the mass, height, age, and gender of the subject, including the walking speed in the predictors is required for *R* > 0.7.

Although the inclusion of knee abduction-adduction angle in the predictors generally improved the prediction of KJCF peaks in the lateral compartment, its effects on the prediction of medial and summed (non-compartmental) KJCF peaks were inconclusive, sometimes increasing prediction accuracy and sometimes reducing it. When comparing predictor sets *P*_*B*_ and *P*_*D*_, the summed loading response peak was the only response variable that failed to improve by the inclusion of knee abduction-adduction angle.

In summary, Table 4 shows that summed (non-compartmental) KJCF peaks were predicted with better accuracy than compartmental peaks. Full-stance peaks and terminal extension peaks were predicted with *R* > 0.5 to *R* > 0.7 and more accurately than loading response peaks. Walking speed was an important predictor of all KJCF peaks, while knee abduction-adduction angle was beneficial in predicting lateral peaks.

No prediction algorithms with meaningful prediction accuracy could be trained to predict MFRs using the predictors in this study, which is why MFR responses are not included in the tables.

## Discussion

Using musculoskeletal (MS) modeling, we analyzed motion capture data from 290 subjects and over 5000 walking trials to retrieve the time series of knee joint contact forces (KJCFs) during different parts of the stance phase and separately in the medial and the lateral compartment. We then trained feedforward artificial neural networks (ANNs) to predict KJCF peaks from input data that can be collected without laboratory-grade motion capture (MOCAP). Our ANN models, which have only one hidden layer with relatively few nodes, do not require MOCAP data, and predict only the maxima of joint loading, can in best cases reach Pearson correlation coefficients above 0.8 (Table 4, top left and bottom left). We achieved such results when walking speed was included in the predictors in addition to demographic and anthropometric predictors. While previous studies have also used ANNs or other machine learning algorithms to predict KJCFs [2, 4, 12, 20, 28] and eliminate the time-consuming MS modeling steps, they still utilized varying amounts of MOCAP data. Our results are a promising step toward predicting KJCFs with simple subject characteristics.

During MS analysis, we observed that the typical tibiofemoral KJCF loading curve over a stance phase had two distinct maxima: the loading response peak and the terminal extension peak. The terminal extension peak was often higher than the loading response peak. When identifying response values in such trials, the terminal extension peak was also the full-stance peak, which explains why the full-stance peaks and the terminal extension peaks resulted in similar prediction accuracies (Table 4). When the subject walked slowly, the typical loading curve distorted as especially the loading response peak became flatter, in some cases merging into the ascent toward the terminal extension peak. Consequently, the loading response peaks either varied a lot or could not be identified in many low-speed trials. Therefore, the data available for predicting loading response peaks were less comprehensive and noisier than that for terminal extension and full-stance peaks, and the prediction accuracy of loading response peaks was smaller. The high prediction accuracy of terminal extension and full-stance peaks can be at least partially attributed to this effect, as the terminal extension peaks of the loading profile underwent more predictable changes with changing walking speed. In the context of KOA studies, difficulties in measuring loading response peaks at low walking speeds are undesirable because pain may cause KOA patients to walk slower than healthy subjects. Alternative derived measures of KJCF, such as area under the curve or mean loading, could perhaps be predicted more accurately than peaks for the loading response phase but were outside the scope of our study.

In general, the summed peaks were predicted with better accuracy than the compartmental peaks (Table 4) and this was expected. Because the summed peaks have a greater magnitude than compartmental peaks, absolute prediction errors in summed peaks do not affect the Pearson correlation coefficient as strongly as the same errors would for compartmental peaks.

Because the inclusion of knee abduction-adduction angle had only small impact on prediction accuracy and the changes were often within the standard deviation of the accuracy measure (Table 4), we cannot draw final conclusions about its effect. The angle was measured during static standing, so it seems reasonable to assume it is also present during the stance phase and affects the KJCF distribution between the medial and lateral compartments. Our choice to lock the knee abduction-adduction angle of the subject-specific model during MS analysis to zero (rather than setting it to whatever was estimated for the subject based on static standing trials) reduces the importance of the angle as a predictor of mediolateral load distribution. Our choice was based on validating the method against in vivo data (Fig. S1). However, because the difference between locking the angle in the model to zero or to its estimated value is small, allowing nonzero abduction-adduction angles during MS analysis may be reasonable in future studies.

The inclusion of walking speed in the predictor set improves prediction accuracy for all response variables (Table 4). Intuitively, walking speed should modulate the force impulses the knee joint experiences, so its accuracy-improving effect was expected. The direct proportionality between walking speed and knee joint loading has also been documented in literature; in 2020, Giarmatzis et al. showed that, with increasing walking speed, the loading response and terminal extension peaks increase in both joint compartments [12]. Additionally, Brisson et al. [4] found that walking speed correlated with medial loading response peaks. Furthermore, Bergmann et al. have shown with total knee replacement patients that in vivo joint loading is greater during jogging than walking [3], although in their study there are likely several factors instead of only locomotion speed involved.

Because the RMSEs of our ANNs (Table 4) are in the same scale as the mean ± standard deviation of the standard deviations of response variabilities (Table 3), the ANNs generalize well and without substantial underfitting or overfitting. Therefore, the ANN architecture we used could also be viable in similar prediction studies of biomechanical functions. This observation is important because although the universal approximation theorem states that an ANN can approximate KJCF peaks, there is no guarantee that the hyperparameters we selected can facilitate such a network. In this study, we showed that they can.

Comparison of accuracy measures with previous studies is difficult because of differences in, e.g., predictor variable selection and validation schemes. To our knowledge, no other study has predicted KJCF peaks or KJCFs using our predictors without including GRFs or marker trajectories in the predictor variables. In the context of our study, it is important to note that we trained and evaluated our ANN so that there was no subject overlap between the training, validation, and test subsets (however, overlap of original datasets was allowed). The ANN prediction accuracies of previous studies can reach Pearson correlation coefficients above 0.9 when MOCAP data are included in the predictors [2, 20]. Our ANN models achieved Pearson correlation coefficients above 0.8 without MOCAP data.

Even though our prediction results were promising including time series information of gait in the predictor set could enable the prediction of the entire stance phase KJCF time series, as has been done with many previous studies [2, 20, 28, 30]. Including time series does not necessarily mean returning to a cumbersome motion laboratory because with existing solutions, such as OpenPose [6], a video camera is sufficient to obtain some gait data [26]. An interesting topic of future research is how computer vision can be integrated into our method to include time series data from, for instance, knee flexion-extension angles to enable KJCF time series prediction while retaining the simplicity of the method. Information about the geometry of the knee joint could also be a valuable addition to the predictor set. Although our musculoskeletal analysis pipeline involved subject-specific scaling of the intercondylar distance based on subject height (see supplementary material), no direct information of knee geometry was included in the predictor set. Although such information could improve predictions of compartmental KJCF maxima and subject specificity of the ANNs, collecting the information would require MRI or X-ray scans, which require specialized equipment and operators to obtain. Such equipment is often found in clinical settings and including knee geometry in the predictors is a potential future research direction. We acknowledge that our method is highly simplified because it predicts peaks of KJCF curves rather than the entire curves and relies on predictors that are easily collected rather than being the best variables to predict KJCF.

Assessing the validity of OpenSim-derived KJCF peaks is difficult because different sources have been shown to have greatly varying results with respect to the bodyweight (BW) of the subject [8] and because available in vivo data is limited. After gathering existing studies, Fregly et al. summarized that maximum KJCF peak ranged from 1.8 to 3.0 BW for forces measured in vivo and from 1.8 to 8.1 BW for MS modeling estimates [10], while D’Lima et al. summarized KJCF peaks from 2 to 3 BW for in vivo forces and 1.7 to 7 BW for MS modeling estimates [8]. Additionally, even though we validated our analysis pipeline against in vivo measurements, the in vivo dataset had only six subjects [31]. Nonetheless, Figure S8 shows that with a median of 3.15 BW, 25th percentile of 2.79 BW, and 75th percentile of 3.59 BW, our MS modeling estimates of summed full-stance peaks fit within the range for MS modeling estimates presented by Fregly et al. Therefore, our MS analysis outputs mostly conform to previous literature.

This study was subjected to limitations that need to be addressed. First, the weights for training samples can be formulated in different ways. However, in preliminary training runs of the ANNs, compared to using no training weights (i.e., equal weights for all data samples), our weights set had little effect on the prediction accuracy of the ANNs. We did not report the difference in prediction accuracy with and without weights, so we cannot quantify the role of weights in loading prediction and whether differently formulated weights have a significant effect on knee loading maxima or MFR prediction should be investigated in later studies.

Second, because subjects with masses above 100 kg came only from the CAROT dataset, the prediction results for heavy subjects contain bias from just a single dataset and, therefore, should be evaluated critically. Additionally, it should be noted that subjects in the CAROT dataset were diagnosed with KOA and we ignored the severity of the KOA, which could be important to consider in formulating the training weights.

Third, in addition to mass, limited overlap between original datasets exists in other predictors. These predictors include walking speed (where both the lowest and the highest values are represented by subjects in the Schreiber dataset), knee abduction-adduction angle (CAROT dataset has the largest range but proportionally the least number of values close to zero), and age (particularly in the 40 to 50 range, which is represented only by subjects in the Schreiber dataset). To improve generalization of the prediction models and mitigate dataset-specific bias, we require a lot of overlap in predictor values between the original datasets. In fact, although we had many subjects and samples, due to differences in subject preparation and marker placement, a greater number of different datasets are required to improve generalization.

Fourth, the original datasets analyzed in this study contained barefoot trials, but in daily life much of walking is done while wearing shoes or similar footwear. The influence of footwear on foot biomechanics has been previously studied [33] and GRFs, joint kinematics and joint kinetics have been found to vary between barefoot and shod walking. Therefore, it would be ideal to have datasets with gait data from shod subjects. However, wearing footwear can make it difficult to perform biomechanical analyses, and additionally, footwear type has been shown to affect the biomechanics of gait [33], which would make it difficult to standardize shod gait trials. Thus, barefoot gait trials provide a standardized way to analyze gait biomechanics and we must assume that the same relationships between predictors and knee loading exist in both barefoot and shod walking.

Fifth, if predictors include gait speed and knee alignment that are calculated from markers, then the models using those predictors require motion capture (MOCAP). However, while in this study we calculated those parameters from MOCAP data, MOCAP is not required to obtain them, as gait speed can be approximated with a stopwatch and a tape measure and knee alignment can be measured with a goniometer, to name simple examples (although, e.g., with goniometer accurately locating the hip joint may be difficult). The predictors obtained that way will slightly differ from the predictors that were used to train the models due to the different methods to measure the predictors. For example, Handa et al. [16] validated stopwatch measurements of walking speed to marker-based optical motion capture and found high correlation (*R* > 0.9) between speeds measured with stopwatch and motion capture, although they also noted that the observed speeds were influenced by the operator of the stopwatch. Furthermore, Oh et al. [27] compared stopwatch-based walking speed to an automatic ultrasound-based timing system and found that the stopwatch overestimated walking speed when the subject was already in motion in the beginning of the measurement. Quantifying the error and the correlation between different measurement methods of the same predictor is an interesting topic for future studies. Therefore, we cannot yet quantify how well knee alignment and walking speed measured with simple methods in a clinical setting correlate to our predictors. Additionally, because including frontal plane knee angle in the predictor set had inconclusive effects on prediction accuracy, omitting it from the predictors in future studies is a possibility if it cannot be effortlessly measured.

Sixth, the ANN prediction accuracy results may be biased by the fact that we visually checked MS-estimated KJCF curves and excluded some trials before constructing the combined dataset and training and validating the ANNs. While the trials we excluded did not represent successfully captured or natural walking (because of, e.g., the presence of only one half of stance phase, marker artifacts resulting in unrealistic kinematics), such artifacts sometimes occur during motion capture sessions; therefore, the ANNs are trained on the features present in successful rather than all motion capture trials. Furthermore, the fact that a single person did the visual validation may introduce small bias into the ANNs. However, the reason we excluded erroneous trials in the first place was to avoid bias causing prediction of biomechanically invalid peak values.

In conclusion, we took promising steps toward predicting knee joint loading peaks during gait without requiring measurements in a motion laboratory. This could enable knee joint loading prediction in environments, such as during clinical examination, eliminate time-consuming analysis steps, and enable the operator to immediately view the results. In future, this method may offer a significant improvement for the clinically applicable prediction models of knee osteoarthritis as those models currently rely on generic loading inputs based on the body weights of the subjects [21, 24, 25]. However, it should be noted that optimal ANN models should be trained on larger training datasets, which would consider all possible variations in subject characteristics (such as age, height, weight, type of knee injury).

### Supplementary Information

Below is the link to the electronic supplementary material.

Supplementary file1 (PDF 1879 KB)
