# Supplementary material for: Prediction of Knee Joint Compartmental Loading Maxima Utilizing Simple Subject Characteristics and Neural Networks
Source: Ann Biomed Eng. 2023 Jun 19;51(11):2479–89. doi: 10.1007/s10439-023-03278-y (PMC10598099; doi:10.1007/s10439-023-03278-y)
Supplement: Supplementary file 1 — Supplementary file1 (PDF 1879 KB) [file 10439_2023_3278_MOESM1_ESM.pdf]

Supplementary material

Musculoskeletal modeling and simulation to estimate knee joint loading

The musculoskeletal modeling pipeline utilized in the current study was optimized and validated for estimating tibiofemoral contact forces before the final analyses of the dataset. Details on the process of optimization and validation can be found from *Validation of the musculoskeletal modeling and simulation* section of the Supplementary material.

Musculoskeletal modeling and simulation was performed using the open-source musculoskeletal simulation and analysis software, OpenSim (version 4.1), [3]. A generic musculoskeletal model designed for simulating human gait [15], with 80 muscle-tendon actuators at the lower limbs, was modified by removing the arms and adding their mass to that of the torso and by including a knee mechanism developed by Lerner et al. [11]. The knee mechanism allowed partitioning of the total tibiofemoral contact force to the forces transmitted through the medial and lateral compartments of the knee. We locked subtalar and metatarsal joints due to insufficient marker sets for detailed analysis of foot motion. The resulting model had one degree of freedom (DoF) ankle joint, one DoF knee joint, three DoF hip joint and six DoF pelvis-ground joint. In addition, the model had three DoF lumbar joints but the torso segment kinematics were not estimated due to some of the datasets missing markers on the upper body and thus the torso was included in the model just for visual purposes.

The analysis pipeline included the following steps: experimental data extraction, creation of subject-specific musculoskeletal model by scaling the generic model, calculation of inverse kinematics (IK) to find generalized coordinates (joint angles), inverse dynamics (ID) to calculate

net intersegmental forces and moments, static optimization (SO) to solve individual muscle forces, and finally calculation of joint contact forces using joint reaction analysis (JRA).

In the experimental data extraction marker trajectories and ground reaction forces (GRFs) were read from C3D motion capture files. The stance phase was detected based on the vertical GRF threshold (dataset depending, see details from *Dataset-specific notes in the musculoskeletal analysis workflow*) and further analysis was commenced only for the stance phase. We analyzed a single stance phase per trial. Automated checks were implemented to identify those ground contacts that were made by the leg of interest (more symptomatic leg in the CAROT dataset, dominant leg in the Fukuchi dataset, and right leg in the Camargo, Horst, and Schreiber datasets), verify that the contact with the force plate was made only with a single leg and the foot did not make contact to the ground beyond the force plate.

A static standing trial was used to scale the generic musculoskeletal model and adjust the positions of virtual markers on the model. The markers used for scaling the segments differed between the datasets due to the different marker sets utilized. For the CAROT dataset, which utilized Vicon's Plug-in-Gait marker set and had biomechanical modeling performed on the trials, we utilized joint centers for scaling the segments. For the other datasets, we used markers on easily defined anatomical landmarks. The location of medial and lateral tibiofemoral contact points in the frontal plane was set according to a regression equation that estimated femur intercondylar distance based on participant's body height (see details from the section *Knee intercondylar distance*). During the scaling, we also estimated participant-specific frontal plane knee alignment (varus/valgus angle) to be used as a predictor in the artificial neural networks. This was done by opening the varus/valgus DoF of the knee joint and letting the IK algorithm estimate the joint angles during static standing. Knee alignment determined using this approach has been shown to correlate with

knee alignment measured from radiographs [13,21]. However, the participant-specific knee alignment was not utilized for subsequent musculoskeletal simulations since our validation showed that assuming the knee alignment to be neutral provided a better estimate of the medial-lateral distribution of the tibiofemoral contact forces. Finally, we opted to preserve the mass distribution of the segments during scaling.

After scaling the segments of the model, we scaled the muscles forces of the model by multiplying each muscle's maximal isometric force by a scaling factor based on the following equation:

$$\left(\frac{\text{SUBJECT MASS}}{\text{GENERIC MODEL MASS}}\right)^{\frac{2}{3}}.$$

The muscle strength scaling method has been used previously [7] and follows from allometric scaling of surface area-based properties (muscle strength depends on the physiological cross-sectional area of the muscle).

Joint kinematics during walking were calculated using the IK algorithm which estimates joint angles by minimizing the sum of the squared difference between the positions of the virtual markers of the model and the positions of the experimental markers. The input marker trajectories used was dataset dependent but we favoured markers on clusters when available. When used as input in subsequent analyses, the IK was lowpass filtered with a 12-Hz cutoff frequency. We used the IK and the GRFs as inputs to solve the net intersegmental forces and moments using the ID algorithm. Subsequently, using the SO tool, we solved the contributions of individual muscle forces to the inverse dynamics solution by minimizing the sum of squared muscle activations. The model was supplemented with reserve actuators at each DoF to prevent failing simulations in case the muscles in the model would not be able to provide the required joint moments. The optimal force (joint moment provided with activation coefficient of 1) of these reserve actuators was set to

1 Nm discouraging their utilization during SO. However, the reserve actuator in the hip flexion/extension DoF was defined so that it had 1 Nm optimal force in the extension direction but a 100 Nm optimal force in the flexion direction. Thus, SO favoured using the reserve actuator for providing the hip flexion moment. The decision was based on our validation of tibiofemoral contact force estimation and the findings by Simonsen et al. [18] suggesting that hip flexion moment in walking is, at least partly, provided by passive structures such as ligaments rather than muscle forces. The hip flexor reserve actuator was used to model the effect of passive structures providing the moment.

Finally, we used the JRA tool to estimate the resultant forces and moments transferred between consecutive bodies as a result of all loads acting on the model. From this analysis, we extracted the forces applied to the medial and lateral contact points of the tibia (representing forces transmitted through medial and lateral compartments of the tibiofemoral joint). From these forces, we concentrate on the component of the force along to long axis of the tibia (i.e. component perpendicular to the tibial plateau). In case the lateral compartment contact force reported by the JRA was a physiologically impossible tensile force, we set the force to zero. In this case, it is assumed that the lateral compartment is unloaded and the force required to stabilize the tibiofemoral joint in the frontal plane was provided by the lateral collateral ligament [22]. Total tibiofemoral contact force was calculated as the sum of the medial and lateral compartments.

We refer to the forces calculated for the tibiofemoral joint as knee joint contact forces (KJCFs). The KJCF outcomes that we tried to estimate using artificial neural networks were the loading response and terminal extension peaks (the first and the second peaks of the stance phase, respectively) and the full-stance peak (the higher peak of the first and second peaks) of the medial, lateral, and total (medial and lateral sides summed together) KJCF. We also calculated medial

force ratio (MFR) of the loading response, terminal extension, and total KJCF by dividing the value of the corresponding medial KJCF by the peak of corresponding full-stance KJCF at the instant where the peak occurred. Therefore, we tried to estimate nine KJCF outcomes and three MFR outcomes.

Validation of the musculoskeletal modeling and simulation

Optimization and validation of the musculoskeletal modeling and simulation pipeline was done using CAMS-Knee dataset [19]. The dataset provides direct *in vivo* measurement of three-dimensional forces and moments applied to the tibial component of total knee replacement from six participants. The compressive force applied on the medial compartment was calculated as

$$F_{med} = \frac{-F_z}{2} - \frac{M_y}{l},$$

where F_z is the compressive force applied to the tibial tray of the implant, M_y is the frontal plane moment and l is the distance between the medial and lateral contact points [8]. The dataset also provides motion capture data (marker trajectories and GRF) and we used these to simulate the KJCFs. The simulated KJCFs were compared with the directly measured.

Walking trials with self-selected walking speed were utilized to optimize and validate the simulation pipeline while data from stair descent was used for scaling the musculoskeletal models as it included a phase of static standing. Three to four stance phases of walking were included in the validation from each of the six participants and used for selecting the following parameters for modeling and simulation pipeline of the current study: participant-specific vs. generic frontal plane alignment of the knee, participant-specific vs. generic femoral intercondylar distance, reserve

actuator strength and muscle force scaling. The reasoning for exploring these parameters is briefly explained below.

Frontal plane knee alignment (knee adduction angle) is known to be an important factor that affects the medio-lateral distribution of tibiofemoral contact forces [8]. Measurement of dynamic knee adduction angle during gait is heavily affected by soft-tissue artefacts [1] and thus, it is unreasonable to estimate kinematics of this DoF based on marker trajectories. However, it may improve the estimation of the medio-lateral distribution of tibiofemoral contact forces if the knee alignment is adjusted participant-specifically and kept statically in that alignment for further analyses. In absence of imaging-based knee alignment measurement, the alignment can be estimated based on motion capture data [13,21]. We tested the effect of having the frontal plane knee alignment as neutral (0 degrees of adduction) or as participant specific based on motion capture data and found that the assumption of neutral knee alignment produced a slightly better and more consistent estimate of the medio-lateral distribution of KJCFs (Fig. S1). More specifically, keeping the frontal plane knee alignment neutral produced a more consistent estimate of the MFR in which lateral unloading was not present while all participants presented similar predominant loading of the medial compartment as calculated also from the in vivo data. The mean MFR was also slightly better estimated at the peak of the total tibiofemoral force with neutral compared to informed knee alignment. However, the total KJCF was slightly better estimated in the first half of the stance when knee alignment was estimated using motion capture. It is worth noting that we observed the more consistent MFR estimation using the neutral knee alignment although there was an average of 3.8 degrees of varus alignment in the participants as measured by post-surgery computed tomography [9] and we were able to reasonably estimate the alignment using the motion capture data ($r = 0.82$). We selected to keep the knee alignment neutral for the analyses. However, the alignment

was estimated during the scaling process and the information on the estimated alignment was saved for use in the artificial neural network prediction of KJCFs.

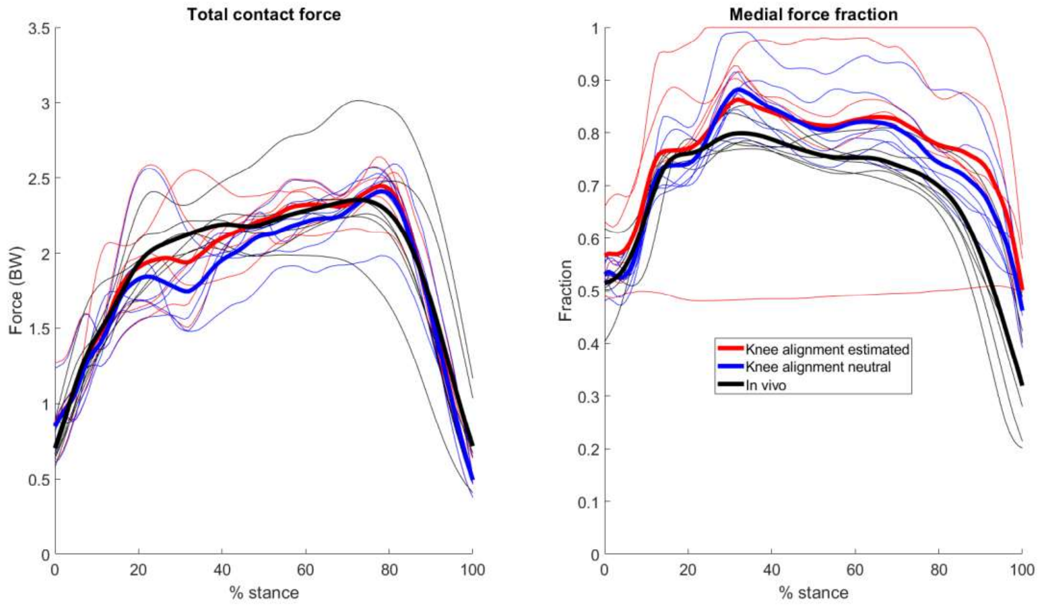

Figure S1. Simulated and measured total tibiofemoral contact forces and medial force fraction (medial force ratio) with participant-specifically estimated and neutral frontal plane knee alignment. The thin lines represent the ensemble average of the simulated stance phases for each participant and the thick lines represent the average across participants. All other model parameters were set as in the final selected pipeline other than the knee alignment.

The distance between the medial and lateral tibiofemoral contact points in the implemented knee model affects the medio-lateral distribution of tibiofemoral contact forces [11] and hence may be important to set them participant-specifically when possible. We tested the effect of this by running the analyses with a model having the intercondylar distance estimated based on a regression equation with the distance between the markers over the medial and lateral epicondyles of the femur as the input [22] and with a model having the intercondylar distance set based on known

implant geometry. The intercondylar of the implant was found by measuring the distance between the apex points of the convex surfaces of the femoral implant geometry. The tibial tray had a corresponding concave shape and thus it was assumed that the center of pressure on both compartments is located on these points. It was found that the model with intercondylar distance informed by implant geometry produced a better estimate of the medio-lateral distribution of tibiofemoral contact forces, especially in early stance (Fig. S2). The average intercondylar distance obtained using the regression equation was 73 mm for this population which is considerably longer than typically observed (see Fig. S5). This difference is potentially due to differences in the populations and the amount of soft tissue over the epicondyles. Since in this study we had participants with varying body compositions (from lean to obese) we decided to create a regression equation to estimate the participant-specific intercondylar distance from a measurement that is not depending on body composition (see section *Knee intercondylar distance*) and used this regression equation to set the intercondylar distance for the analyses.

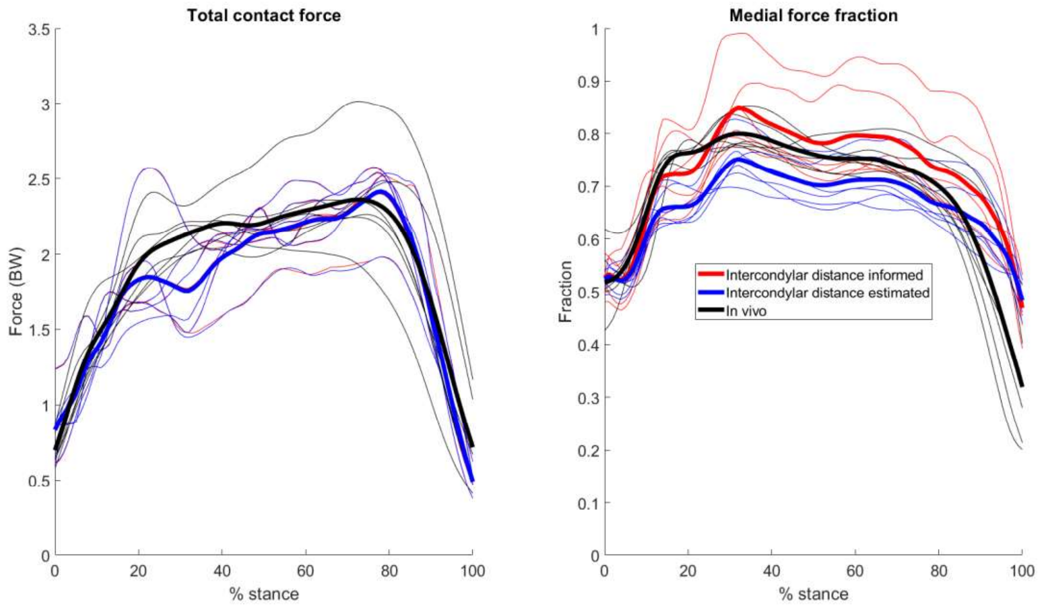

Figure S2. Simulated and measured total tibiofemoral contact forces and medial force fraction (medial force ratio) with informed and estimated intercondylar distances of the knee. The thin lines represent the ensemble average of the simulated stance phases for each participant and the thick lines represent the average across participants. All other model parameters were set as in the final selected pipeline other than the intercondylar distances.

Preliminary analyses with the CAMS knee dataset indicated that the second peak of the simulated tibiofemoral contact force may be overestimated. The overestimation was associated with a high rectus femoris muscle activation during the late stance when the internal hip flexor moment occurs. This causes a cascade of compensatory muscle activations in hamstrings and gastrocnemius to overcome the knee extension moment that the rectus femoris creates while flexing the hip. Simonsen et al. [18] concluded based on their experimental findings that the hip flexor moments in late stance is mainly due to passive elastic structures resisting hip joint extension. As typical musculoskeletal models used for simulating walking, also the one utilized in the current study, does not model passive structure around the joints, the muscles in the model are required to

generate the moments that those structures create. In the case of hip flexor moment, this limitation in the model may cause the overestimation in the second peak of the tibiofemoral contact force. To overcome the limitation we tested if supplementing the model with a strong reserve actuator in flexion direction would improve the accuracy of the simulated KJCFs. The rationale here is that when the model has a strong hip flexor reserve actuator the optimization favours using the reserve actuator to provide the requited hip flexion moment which in turn would prevent potentially unrealistic activation of the biarticular hip flexor, rectus femoris. Thus, the strong reserve actuator in the model can be viewed as a representation of the moments provided by the passive structure around the hip. It should be noted that this approach would not be appropriate if estimating loading of the hip joint. Our analyses showed that the accuracy of the estimated peak of the total KJCF in the second half of the stance improved when the optimal force of the hip flexor reserve actuator was >10 Nm. A value of 100 Nm provided a slightly better estimation of the total KJCF at mid-stance and a slightly better MFR in the second half of the stance than 1000 Nm. Thus, we selected the value of 100 Nm for the analyses of this study. However, it should be noted that the individual responses to the reserve actuator strength were highly variable. Here, we selected the option that provided the best mean prediction. Future studies may utilize other solutions such as EMG informed simulations of modeling the passive structures around the hip.

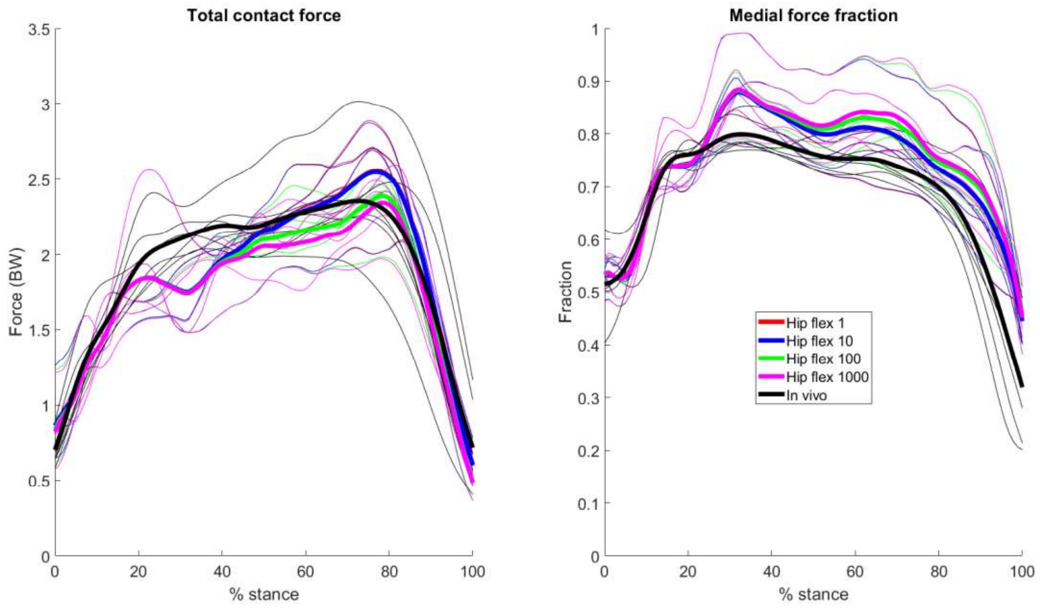

Figure S3. Simulated and measured total tibiofemoral contact forces and medial force fraction with different optimal forces of the hip flexion reserve actuator. The thin lines represent the ensemble average of the simulated stance phases for each participant and the thick lines represent the average across participants. All other model parameters were set as in the final selected pipeline other than the reserve actuator optimal forces.

Muscle strength of the model, i.e. maximal isometric muscle force of each muscle, needs to be adjusted since the body masses vary a lot between individuals in the dataset and differ from the generic model. In lack of any other relevant information, the muscle strength was adjusted by scaling with the assumption that muscle strength adapts to the habitual loading of the body which is dominated by the loading induced by body mass during daily activities. Therefore, a person with higher body mass is assumed to also have stronger muscles [4]. We tested the following scaling factors for the muscle strengths:

$$\text{SCALE FACTOR 1} = \frac{\text{SUBJECT MASS}}{\text{GENERIC MODEL MASS}}$$

$$\text{SCALE FACTOR 2} = \left(\frac{\text{SUBJECT MASS}}{\text{GENERIC MODEL MASS}}\right)^{\frac{2}{3}}$$

$$\text{SCALE FACTOR 3} = \left(\frac{\text{SUBJECT MASS}}{\text{GENERIC MODEL MASS}}\right) * 1.5,$$

which represent, respectively, simple scaling based on body mass, allometric scaling, and scaling based on body mass but all muscle forces increased by 50% to make sure that muscle activations do not saturate to maximum muscle activation. The strength scaling had only a minor effect on the estimated KJCFs (Fig. S4). This was expected since in a task requiring submaximal muscle forces the differences in muscle strength between models can be compensated by adjusting muscles activation during SO. However, we observed a more marked overestimation of the total KJCF at the second half of the stance when the scale factor was set to option 3. We selected option 2 for its strong theoretical basis (allometric scaling of a variable that is dependent on the surface area, in this case, the physiological cross-section area of the muscle) and experimental confirmation in lean individuals [4].

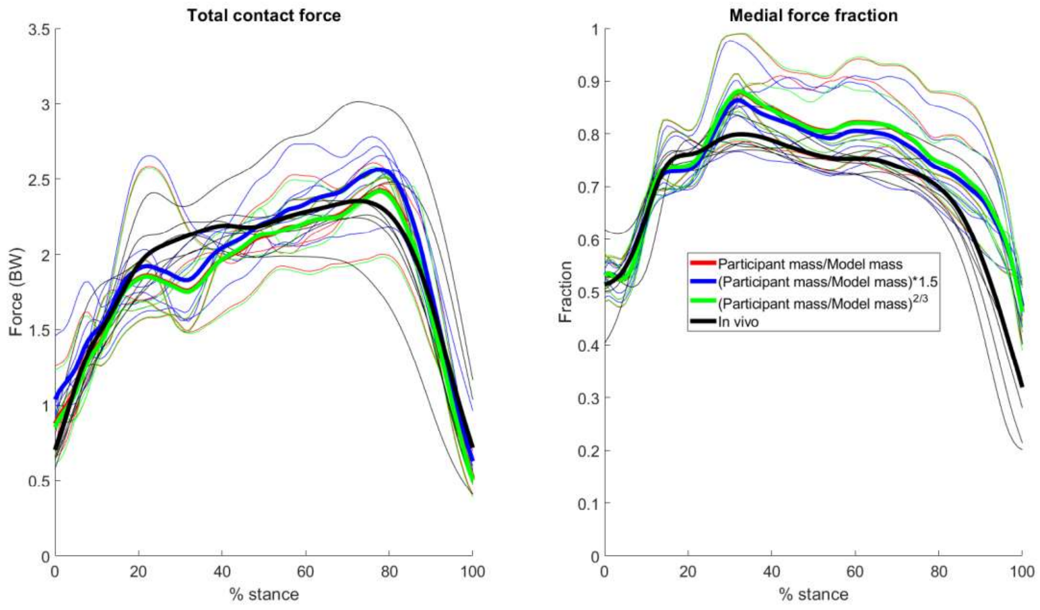

Figure S4. Simulated and measured total tibiofemoral contact forces and medial force fraction with different scaling factors used for scaling the maximal isometric muscle forces (i.e. muscle strength) of the models. The thin lines represent the ensemble average of the simulated stance phases for each participant and the thick lines represent the average across participants. All other model parameters were set as in the final selected pipeline other than the muscle strength scaling.

Knee intercondylar distance

Tibiofemoral contact forces are transmitted through two contact points in the musculoskeletal model utilized in the current study. The location of the contact point in the frontal plane affects the distribution of the contact forces between the medial and lateral compartments of the tibiofemoral joint [11] and hence it is reasonable to set them participant-specifically when possible. A regression equation has been previously established to estimate the femoral intercondylar distance based on the distance between markers placed over the femoral epicondyles [22] and utilized to set the intercondylar distance (ICD) for musculoskeletal models to examine compartmental loading in the tibiofemoral joint [16]. However, this previously established regression equation is critically

dependent on the soft tissue thicknesses around the knee. As this study included participants with varying levels of adipose tissue, we created a regression equation to estimate ICD based on body height. We measured ICD (distance between centre points of each condyle) from magnetic resonance images of 50 participants from the CAROT dataset and obtained a positive correlation between the participants' body height and ICD ($r = 0.584$, $p < 0.001$). The correlation between tibia or femur lengths and ICD were also tested but found to be weaker than the correlation between body height and ICD. The regression model (Fig. S5) was used to set the intercondylar distance for the participant-specific musculoskeletal models in the current study. Similarly, to previous studies, it was assumed that the medial and lateral contact points of the tibiofemoral joint are located equidistant from the knee joint centre [16,22]. Thus, both contact points were located ½*estimated ICD from the knee joint centre in the frontal plane.

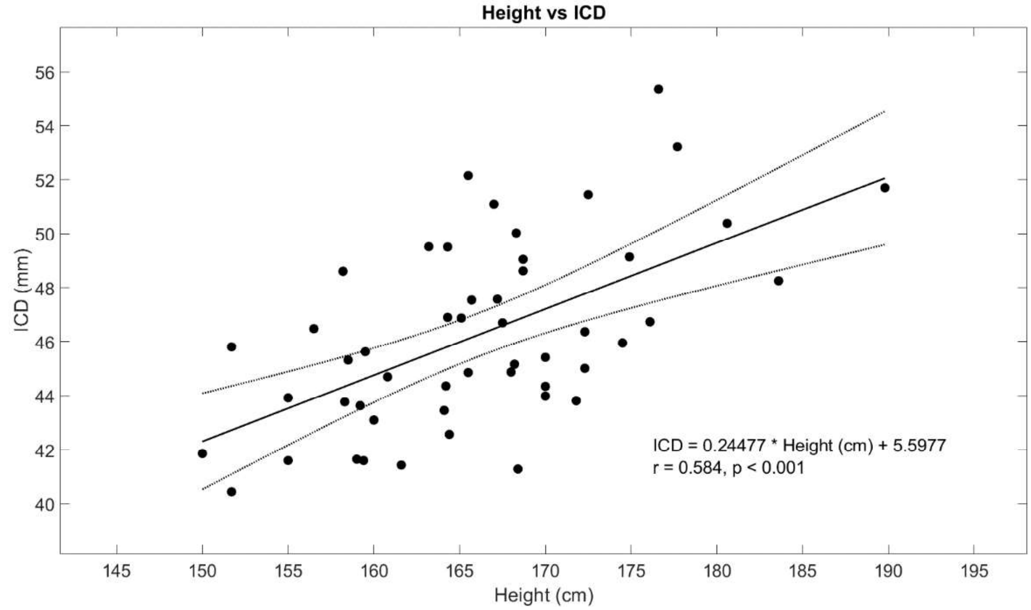

Figure S5. Regression model estimating knee joint intercondylar distance (ICD) from participant's height. The solid line represents the estimated regression line and dotted lines the 95 % confidence intervals of the regression line.

Automatic KJCF peak detection

The KJCF time series in lateral and medial compartments, as well as in their summed profile, is characterized by two maxima resulting from the loading response and terminal extension phases and a local minimum between them. We identified the local minimum by first assuming its location as the minimum between 40% and 60% of the KJCF time series. If we detected the minimum at the edge of this range, the range was broadened until the minimum was detected inside the range. There was generally no need to broaden the range because the local minimum was easily detected.

Loading response and terminal extensions peaks were then defined as the maximum values of the time series on the left and right side of the local minimum, respectively. In some datasets, artefacts were detected as sharp peaks in the beginning or the end of the time series, which could in some cases have values higher than the peaks of interest. To avoid identifying these artefacts as loading response and terminal extension peaks, we used the following algorithm: whenever the maximum was found within a set number of percentage points of the edge of the range (between the beginning of the time series and the local minimum for the loading response peak, and between the local minimum and the end of the time series for the terminal extension peak), the range was narrowed from that side by one percentage point and the maximum was detected again. We used three percentage points as the threshold for loading response peaks and eight percentage points for the terminal extension peaks.

After these detection steps, if we still failed to detect the peaks or the local minimum, the resulting invalid or undefined peak values were set to -1. We then omitted data points with peak values of -1 when the dataset was constructed for artificial neural network analysis.

Dataset-specific notes in the musculoskeletal analysis workflow

The subjects in all datasets performed the walking trials barefoot. However, varying levels of noise and artefacts in the GRFs and marker trajectories, as well as nonstandard marker sets in different datasets, necessitated the use of different parameters in identifying the stance phase, filtering the data, and determining the settings for musculoskeletal simulation-based analyses. Some data may have been filtered twice, but the effect of repeated filtering on the data is negligible.

CAROT

The force threshold for stance phase detection was set to 10 N and the GRF data was lowpass filtered with a 4th order zero-lag Butterworth filter at 12 Hz in Vicon Nexus. The marker trajectories were smoothed as part of the plug-in gait pipeline in Vicon Nexus with a Woltring filter.

Schreiber

The force threshold for stance phase detection was set to 10 N. The GRF and marker trajectory data in the dataset were already lowpass filtered at 15 Hz and 6 Hz, respectively [17]. Therefore, we applied no marker trajectory filtering but filtered the GRF data at 12 Hz.

Fukuchi

The force threshold for stance phase detection was set to 200 N and the GRF data and marker trajectories were lowpass filtered at 12 Hz using a 4th order Butterworth lowpass filter. A large force threshold was necessary to limit the stance phase because otherwise, the lowpass filtering would have caused strong artefacts to COP signal near the time boundaries of the stance phase.

Horst

The force threshold for stance phase detection was set to 300 N and the marker trajectories and GRF data were lowpass filtered at 12 Hz using a 4th order Butterworth filter. Force and moment data were filtered for the whole trial, but COP data were filtered only for the detected contact period.

Camargo

The force threshold for stance phase detection was set to 50 N and both the GRF data and marker trajectories were lowpass filtered at 12 Hz using a 4th order Butterworth filter. There were no separate standing trials in the dataset but scaling was performed on a static standing phase in each walking trial.

Dispersion of response variables in the combined dataset

The values of different response variables and their standard deviations in the combined dataset are presented in Figure S6, where the height of the bar indicates the mean of the response variable and the whiskers indicate its standard deviation. In Figure S7, the range of the values and the 25th and the 75th percentiles are shown, while Figure S8 illustrates the values normalized to the bodyweights of the subjects; the central mark denotes the median value, the edges of the box the 25th and 75th percentiles, and the whiskers the range of all values. Full-stance summed bodyweight-normalized KJCF peaks have a median of 3.15, 25th percentile of 2.79, and 75th percentile of 3.59.

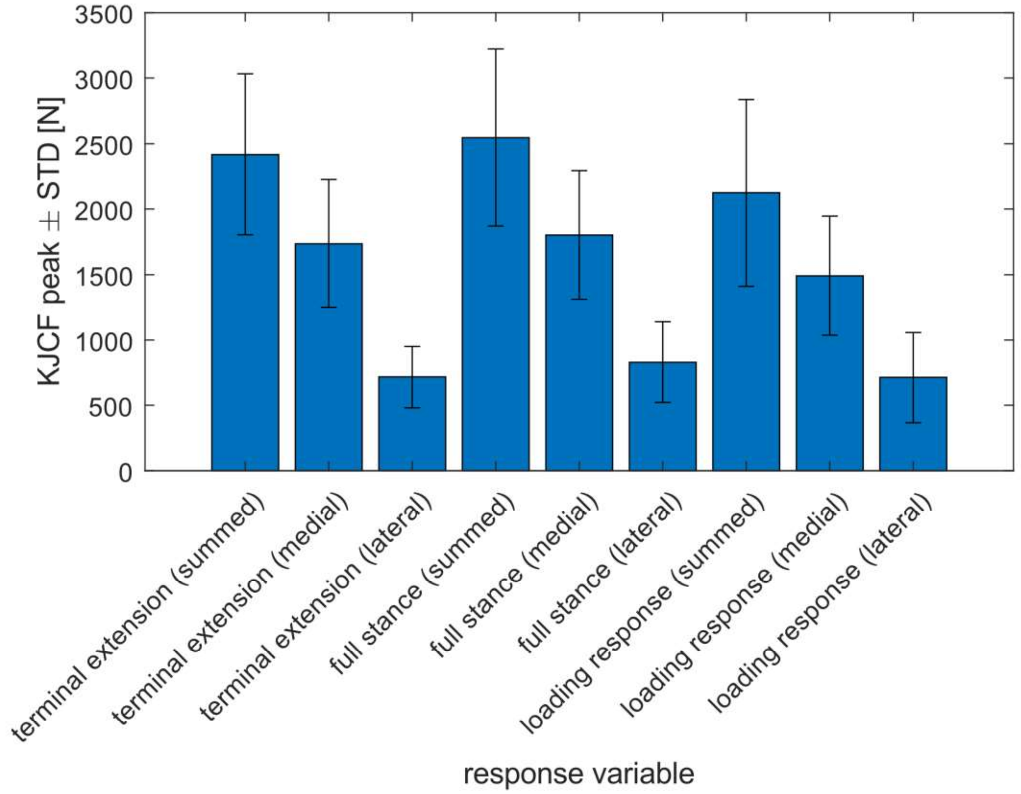

Figure S6. Mean knee joint contact force peaks for different response variables (heights of bars) ± the standard deviation of the peaks (whiskers).

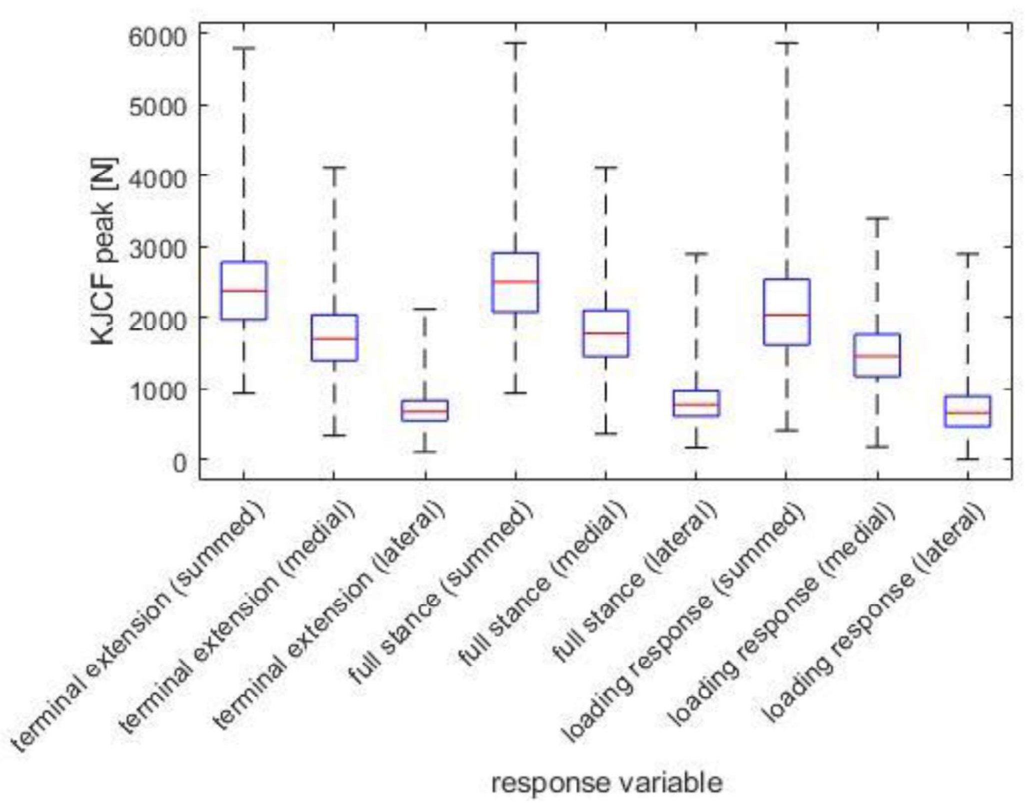

Figure S7. The medians of response values (red marks), the range of response values between 25th and 75th percentiles (boxes), and the ranges of all response values (whiskers).

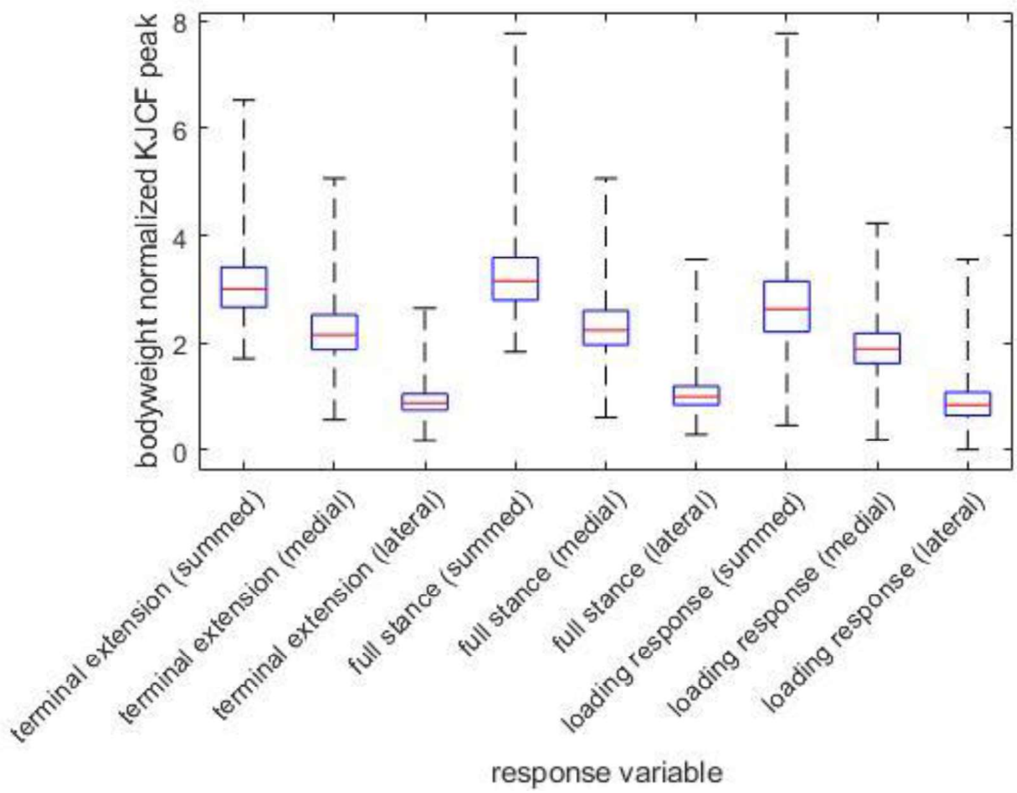

Figure S8. The medians of the bodyweight-normalized response values (red marks), the ranges of normalized response values between 25th and 75th percentiles (boxes), and the ranges of all normalized response values (whiskers).

Nested k-fold cross-validation

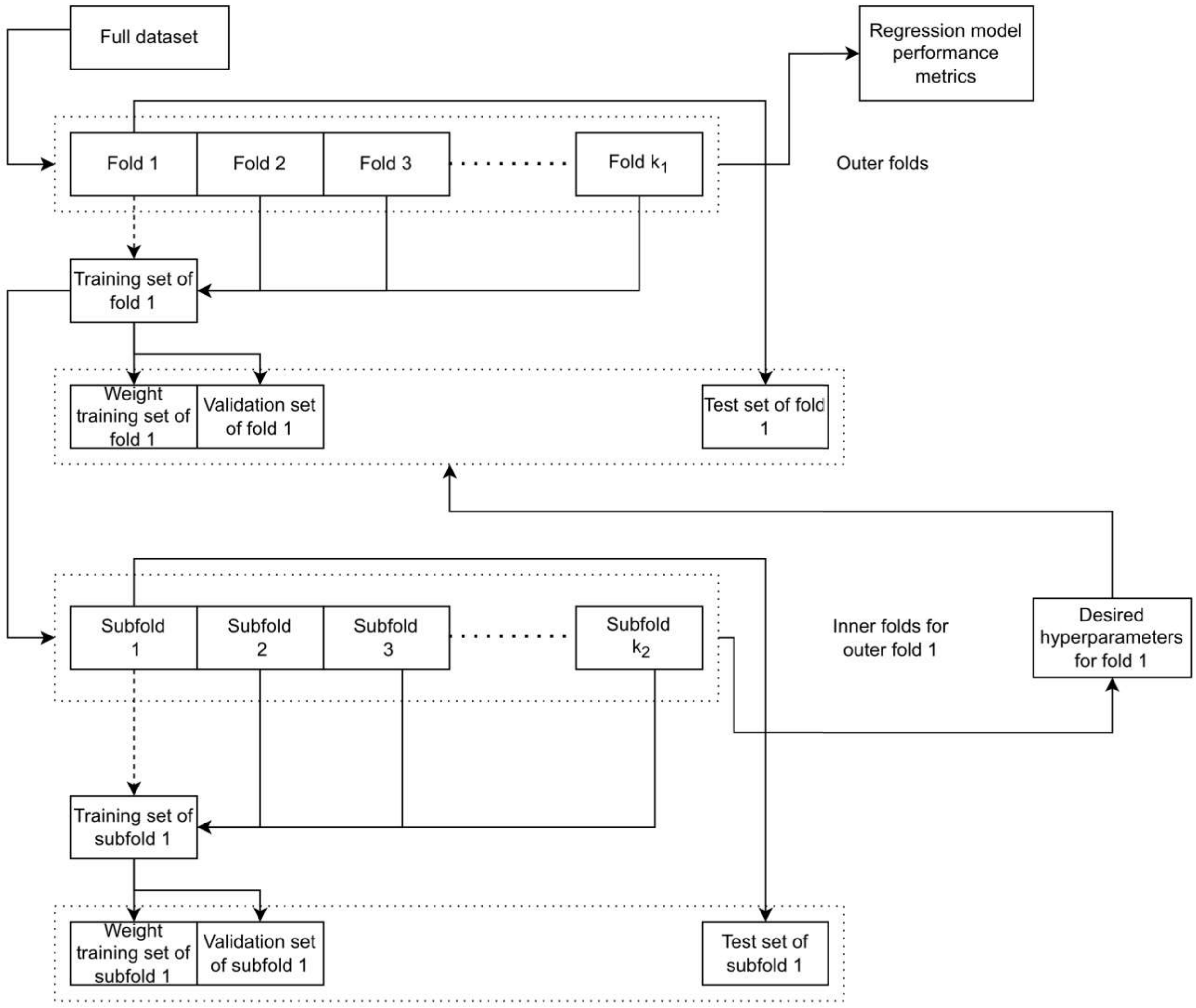

Figure S9. Illustration of the nested k-fold cross-validation algorithm.

Folds in the outer loop

As shown in Figure S9, the full dataset is divided into k_1 cross-validation folds. For each cross-validation fold, we construct a training set and a test set. The training set is constructed from all the data in all folds except the current fold. The data in the current fold then constitutes the test set.

For example, the training set for the first fold (Training set of fold 1) comprises the data from folds 2 to k_1. The test set for the first fold (Test set of fold 1) is just the data in the first fold.

Subfolds in the inner loop

Next, the training set is treated like a whole dataset; for the inner cross-validation loop, it is divided into k_2 folds. Let us call these inner loop folds subfolds to separate them from the folds in the outer loop.

Like in the outer loop, we construct a training set and a test set for each subfold; the data in a subfold is the test set for that subfold, and the data in all other subfolds is the training data for that subfold.

In the illustrated example, data from folds 2 to k_1, also known as the training set of fold 1, are divided into k_2 subfolds; each subfold is assigned a training set comprising the data of all other subfolds (training set of subfold 1), while the data in that subfold is its own test set (test set of subfold 1).

Training and evaluation of inner loop networks

Then, the training set is split into a weight training set and a validation set. The weight training set is used to modify the weights of the artificial neural network iteratively during training; each iteration, the network at that point is used to predict response values in the validation set, and the error is calculated for the validation set. When the error stops decreasing for 6 consecutive iterations, the training stops.

When training stops, the network predicts response values in the test set; the error of these predictions is the error for that subfold. We evaluate that error for all desired hyperparameter combinations. The best hyperparameter combination for each subfold is defined as the combination that that results in the smallest RMSE. In summary, for each subfold and each

hyperparameter combination, we trained a separate artificial neural network and evaluated its accuracy on the data in that subfold.

Training and evaluation of outer loop networks

Now, for each fold in the outer loop, we have k_2 candidates for the best hyperparameter combination. Quite often the candidates are not unique. Therefore, we select the most frequent combination as the hyperparameter combination for each outer fold. As a result, each outer fold has its own hyperparameters, although some folds may share the same hyperparameters.

Similarly to how the training sets of inner folds were split, the training set of each outer fold is split into a weight training set and a validation set. The weight training set is used to train the network, and the validation set is used to determine when to stop training. Lastly, the RMSE for the subfold is evaluated using the test set.

Finally, we take the mean of all k_1 RMSE values to determine the RMSE of the artificial neural network model. Pearson correlation coefficients are processed similarly.

Note that the illustration and the explanation describe a case where early stopping is used with the **trainlm** algorithm. In the case of Bayesian regularization with the **trainbr** algorithm, no validation set exists, and the weight training set is the entire training set.

Formulation of the weights of the artificial neural network training samples

Many trainable algorithms allow the specification of weights for different data samples during training. If no weights are specified, each sample is equally important in constructing the structure of the prediction algorithm. Because the combined dataset contained samples from datasets of different sizes and different subjects within a single dataset had a varying number of trials, it is

justified to specify weights to avoid gross overrepresentation of a single subject, dataset or trial type, which could cause a significant bias.

In many datasets, subjects had trials at different velocities. For example, each subject in the CAROT dataset had trials at a comfortable walking speed and a fixed, instructed walking speed. Therefore, it is reasonable to use weights that give different trial types the same importance regardless of the number of trials within that trial type. On the other hand, if a certain trial type had just one sample and another type had 20 samples, it could be argued that the latter trial type should be weighted more during training because it is less likely to have a high random error in the mean target value due to the high number of repetitive samples.

Additionally, because the trial type structure varied between datasets, creating general weighting rules that apply to all datasets is difficult. However, creating a customized weight-determining algorithm for each dataset would be cumbersome. We devised a satisfactory compromise with four main assumptions that were used to create a general algorithm that created the weights for all datasets.

First, we assumed that initially each subject is equally important in training the prediction algorithm. Therefore, we weighted the trials under each subject so that the sum of the weights equaled 1 for each subject. It made no difference if the subjects had different kinds of trials.

Second, we assumed that the fewer trials there are for a subject, the more random error there is in the biomechanically analyzed KJCF output compared to the actual value. Therefore, we applied a weight to all trials under an individual subject; that weight is a measure of the amount of random error. The measure was chosen as the square root of the number of trials under that individual subject. Now, if a subject had only one trial, it was weighted with a value of 1. If a subject had 4

trials, they were both weighted with a value of 2. If a subject had 16 trials, they were all weighted with 4. This emphasized subjects with many trials over subjects with few trials without completely overriding the effects of the first assumption.

This method has flaws, because in the dataset some subjects had trials where they had been instructed to walk with different velocities. If we had wanted to be more exact, we would have applied the second assumption to these unique combinations of subjects and instructed velocities instead of only subjects. However, we believe that this method was reasonable while being simple to implement.

With the first and the second method combined, if there were N trials under a subject, then each trial was weighted by $\frac{1}{\sqrt{N}}$. Thus, by summing the weights of all trials under a subject, the weight for that subject became $\sqrt{N}$.

Third, we assumed that each dataset except the CAROT dataset is equally important in training the prediction algorithm. Therefore, we wanted to weigh the trials under each dataset except CAROT so that the sum of the weights for each dataset equaled 1. This assumption was like the first, but for datasets with respect to the number of trials instead of subjects with respect to the number of trials. The CAROT dataset, unlike all other datasets included in this study, comprised patients diagnosed with KOA. Therefore, we assumed that CAROT patients' gait only partially represented the gait the ANN should generalize, and the weights of CAROT trials were multiplied by 0.5. This decision was supported by the fact that the gait kinematics of KOA patients have been shown to differ from those of healthy controls [2,14].

Fourth, we assumed that the fewer subjects a dataset has, the less value from variety it has. Therefore, we wanted to give greater weights to datasets with many subjects and smaller weights

to datasets with few subjects. We selected the square root of the number of subjects in the dataset as the weight for the dataset.

For each trial, the weights from these four assumptions were multiplied together to obtain the final weight.

Validation of the artificial neural networks

Between k-fold cross-validation and holdout validation, it is often argued that cross-validation is better if one has the computational resources to use it, because it trains the model and checks it against a test set several times while utilizing the entire dataset for training [5]. This is unlike in holdout validation, where only a portion of the dataset can be used for training and the model is trained and checked against the test set only once [20]. Tohka and van Gils recommend cross-validation for datasets containing less than one thousand subjects [20].

We opted, as per common convention, to use 5-fold cross-validation: the entire dataset is split into 5 folds and each fold is in turn used as the test set to evaluate the prediction accuracy of the network on an independent dataset, while the remaining 4 folds are used to train the network. Furthermore, the data in those 4 folds is split into a training set and a validation set. The training set is used to modify the weights of the network and the validation set is used to find when training should be stopped to avoid overfitting to the training set. After each iteration, the validation set was evaluated in the ANN and its MSE was calculated by comparing the predicted output to the true output. When the validation MSE stopped decreasing for 6 consecutive iterations, the training was stopped to avoid overfitting and the weights at minimum validation MSE were chosen for the final trained ANN. If the training continued to one thousand iterations, it was stopped even if the validation

error continued decreasing. The final test accuracy measures are calculated as the means of the prediction accuracy measures for each test fold [5]. We used 80% of the data samples in the training folds for the training subset and 20% for the validation subset.

Finally, it should be noted that although we treat the test set as an independent set, data samples from any open dataset may be and likely are present in both the training and the test set. Therefore, the same dataset-specific bias may be present in training and test sets. However, we designed the validation algorithm to ensure that samples from an individual subject can only be present in one of the folds (and within the training folds, only in either the training set or the validation set), strengthening our claim of an independent test set.

Our hyperparameter selection procedure is explained in Supplementary materials.

We implemented hyperparameter optimization to select the training function and the number of nodes using nested k-fold cross-validation: another, inner cross-validation loop was implemented inside the outer, main cross-validation loop. The outer loop was used to measure ANN prediction accuracy; the inner loop was used to determine the number of nodes, the activation function in the hidden layer, and the training function for the weights of the ANN that minimize RMSE on the test set of the inner cross-validation loop. Like we did with the outer loop, we chose five as the number of folds in the inner loop. In other words, we performed a grid search on the optimizable hyperparameters in each iteration of the inner loop and picked the hyperparameter combination that minimized test RMSE for evaluating the prediction model in the outer loop.

The nested k-fold cross-validation algorithm was written according to the pseudocode presented by Huttunen et al. [6]. Its implementation was necessary to automatically determine the desired

hyperparameters of the ANN without resulting in biased accuracy measures, as Tohka et al. have shown [20]. An illustrative explanation of the algorithm is provided in the Supplementary materials.

Hyperparameter selection for artificial neural networks

The settings of a machine learning algorithm are called hyperparameters [5]. The hyperparameters of the ANN included the type of the activation functions in the hidden and the output layer, the training function for updating the weights of the ANN iteratively, training function-related parameters that specify how much weights are updated each iteration, the number of hidden layers, and the number of nodes in each layer. Hyperparameters can be determined by evaluating the error of the ANN on a test set for all combinations of different hyperparameters and selecting the combination that results in the smallest error; this process is called hyperparameter optimization. However, its computational cost increases exponentially with each included alternative hyperparameter. Therefore, we selected hyperparameters manually whenever we could justify their selection and used hyperparameter optimization to determine the remaining hyperparameters.

Because KJCF prediction is a regression problem, we chose the linear activation function, also known as the identity function, as the activation function for the output layer. It is suitable for regression tasks because it is unbounded. Thus, it does not limit the final output of the network to a range.

We chose the hyperbolic tangent function (tansig in MATLAB) as the activation function of the hidden layer. In regression problems, it is a common alternative to another very popular activation function, the rectified linear unit (ReLU). However, in preliminary tests we found that the trained ANN was more accurate when we used a hyperbolic tangent function rather than a ReLU, which

is why we settled for the former. For a more thorough explanation of different activation functions, see the MATLAB documentation [12] or a book on artificial neural networks such as [10].

Typically for most regression problems, a single hidden layer is enough. Multiple hidden layers are sometimes used to approximate very complicated functions. We chose a single hidden layer because the function that maps subject characteristics to KJCF peaks is unlikely to be extremely complicated. Our choice is further supported by the fact that our predictor and response variables were scalar values rather than vectors or matrices, so they are extremely unlikely to contain hidden features for multilayer networks to identify. Additionally, provided there are enough neurons in the hidden layer, a single layer is sufficient to fulfill the universal approximation theory [10].

After preliminary testing, we chose the Levenberg-Marquardt (LM) backpropagation algorithm [10] as the training function. It showed the best results in preliminary tests and MATLAB documentation recommended it for nonlinear regression problems requiring very accurate approximation. In MATLAB, the LM algorithm is implemented in two functions: **trainlm** and **trainbr**. The difference in the functions is how they avoid overfitting; **trainlm** uses a validation set with the early stopping method, whereas **trainbr** uses Bayesian regularization. We included both functions in our hyperparameters. Although we utilized both means separately, to avoid needless repetition, we describe our methods as if we used early stopping. Therefore, the reader should note that in the case of regularization, our methods are the same as with a validation set except that no validation set was separated from the training set. Default parameters were used for training function settings.

Preliminary testing showed that in our regression problem, only a few nodes were usually required to reach minimum error. Therefore, we chose the possible number of nodes in the hidden layer to range from one to ten.

Example exclusion cases from the visual validation of KJCF curves

Figures Figure S10 Figure S14 illustrate some KJCF curves that were manually omitted based on visual inspection before the combined dataset was constructed. Reasons include failure of the stance detection algorithm to limit the trial to the stance phase, positive offset in GRFs resulting in unrealistically high KJCF, too weak muscles in the musculoskeletal model leading to inability of the model to generate the required motion, signal distortion in measured moments in force plate data, and marker trajectories jumping in the motion capture data, i.e., measured marker trajectories being momentarily labeled as the wrong experimental marker.

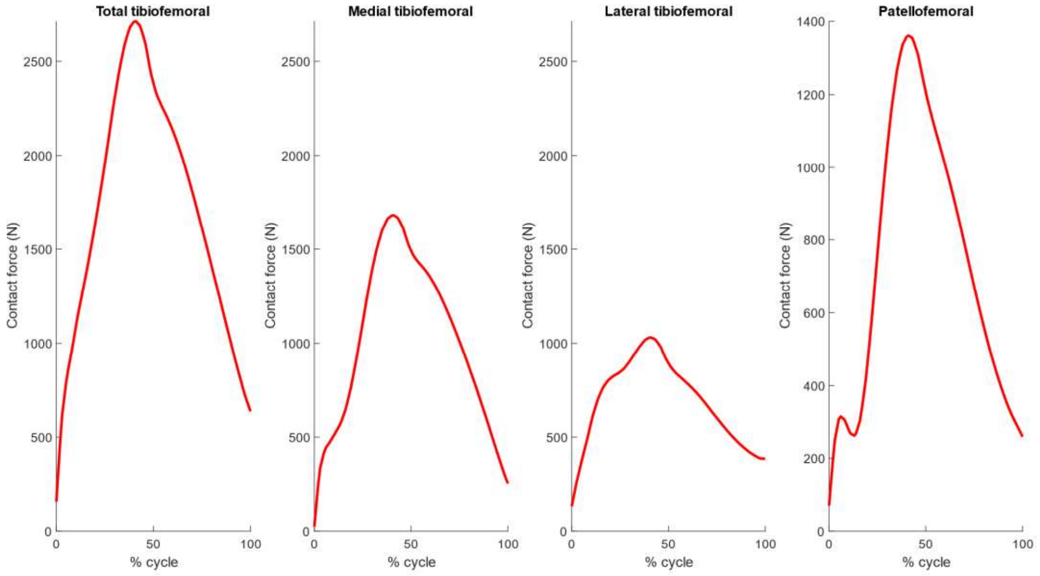

Figure S10. An example of KJCF curves where the automatic stance detection failed to detect the full stance phase and the resulting KJCF curve contains approximately half of the stance phase.

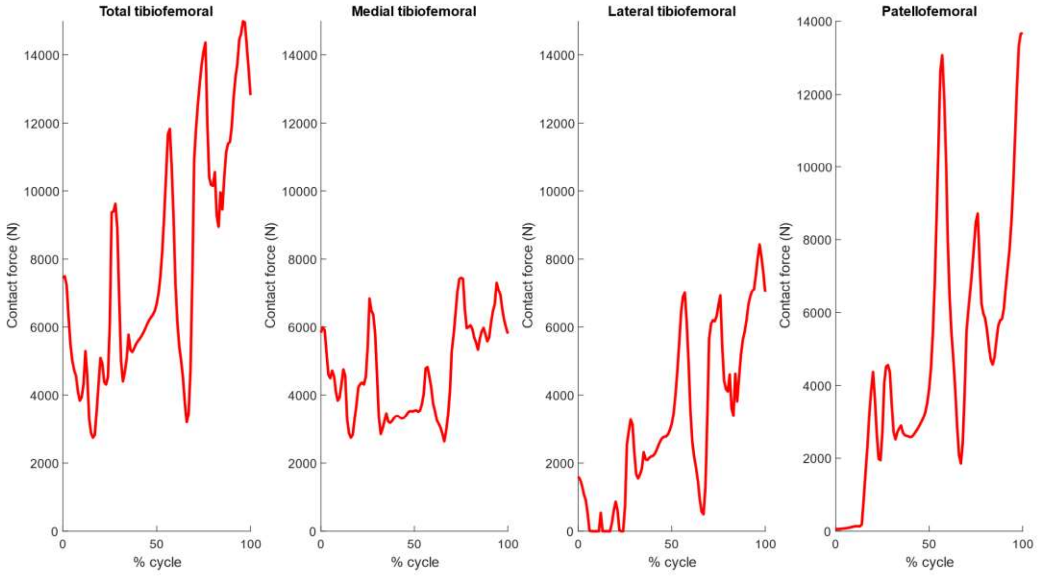

Figure S11. An example of KJCF curves where the automatic stance detection detected several stance phases as one stance, and the force plate forces had an offset artifact.

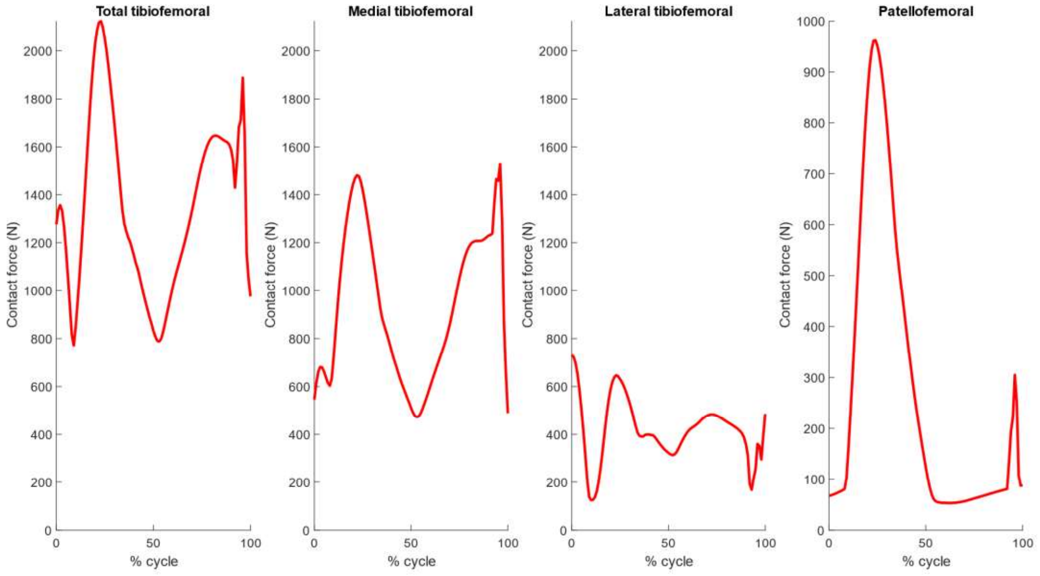

Figure S12. An example of KJCF curves where the static optimization stage of musculoskeletal analysis failed to reach sufficient activation in hip muscles.

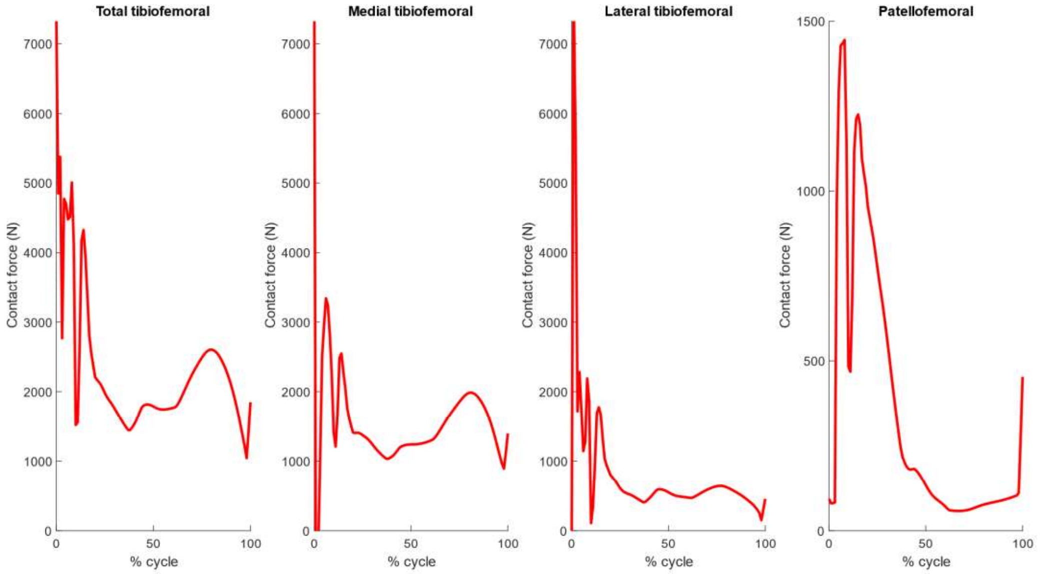

Figure S13. An example of KJCF curves where the force plate moment artifact in the early stance phase distorted the beginning of the KJCF curves.

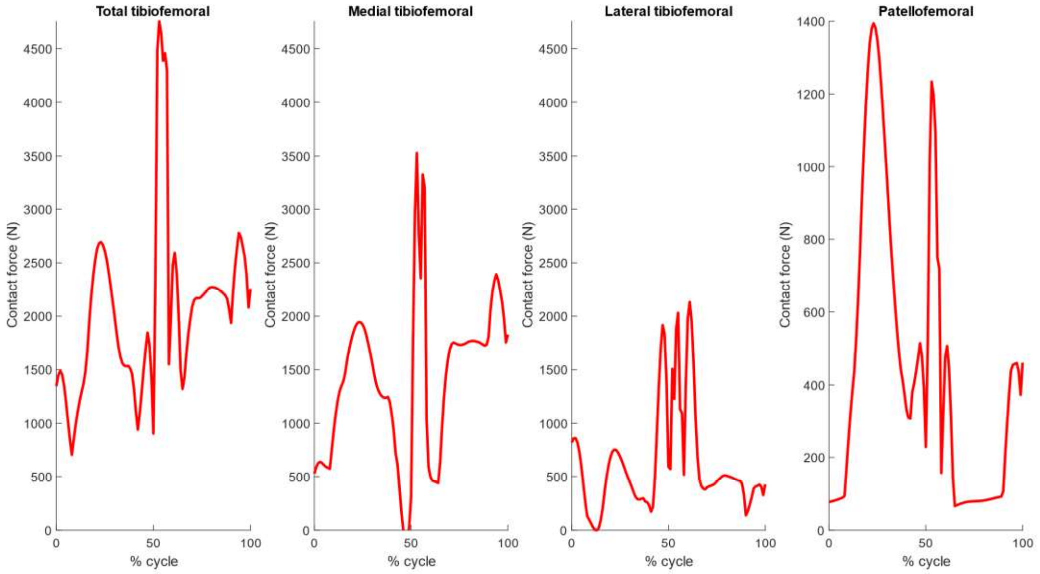

Figure S14. An example of KJCF curves where a single-frame marker jump in motion capture data distorted some joint angles and the middle of the KJCF curves.

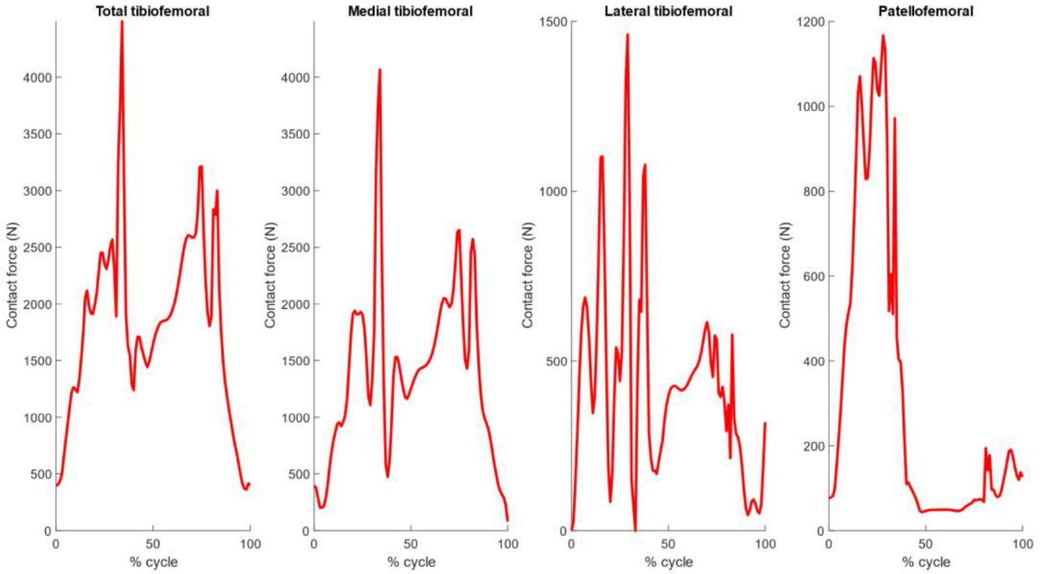

Figure S15. An example of KJCF curves where several marker jumps in motion capture data distorted the KJCF curves.

References

1. Benoit, D. L., D. K. Ramsey, M. Lamontagne, L. Xu, P. Wretenberg, and P. Renström. Effect of skin movement artifact on knee kinematics during gait and cutting motions measured in vivo. *Gait Posture* 24:152–164, 2006.

2. Bytyqi, D., B. Shabani, S. Lustig, L. Cheze, N. Karahoda Gjurgjeala, and P. Neyret. Gait knee kinematic alterations in medial osteoarthritis: Three dimensional assessment. *Int Orthop* 38:1191–1198, 2014.

3. Delp, S. L., F. C. Anderson, A. S. Arnold, P. Loan, A. Habib, C. T. John, E. Guendelman, and D. G. Thelen. OpenSim: Open-source software to create and analyze dynamic simulations of movement. *IEEE Trans Biomed Eng* 54:1940–1950, 2007.

4. Folland, J. P., T. M. Mc Cauley, and A. G. Williams. Allometric scaling of strength measurements to body size. *Eur J Appl Physiol* 102:739–745, 2008.

5. Goodfellow, I., Y. Bengio, and A. Courville. Deep Learning. MIT Press, 2016.

6. Huttunen, H., T. Manninen, and J. Tohka. MEG mind reading: Strategies for feature selection. *Researchgate.Net* , 2012.

7. Van Der Krogt, M. M., L. Bar-On, T. Kindt, K. Desloovere, and J. Harlaar. Neuro-musculoskeletal simulation of instrumented contracture and spasticity assessment in children with cerebral palsy. *J Neuroeng Rehabil* 13:1–11, 2016.

8. Kutzner, I., A. Bender, J. Dymke, G. Duda, P. von Roth, and G. Bergmann. Mediolateral force distribution at the knee joint shifts across activities and is driven by tibiofemoral alignment. *Bone and Joint Journal* 99B:779–787, 2017.

9. Kutzner, I., A. Trepczynski, M. O. Heller, and G. Bergmann. Knee adduction moment and medial contact force-facts about their correlation during gait. *PLoS One* 8:, 2013.

10. Kwon, S. J. Artificial Neural Networks. Nova Science Publishers, Inc., 2011, 1–426 pp.

11. Lerner, Z. F., M. S. DeMers, S. L. Delp, and R. C. Browning. How tibiofemoral alignment and contact locations affect predictions of medial and lateral tibiofemoral contact forces. *J Biomech* 48:644–650, 2015.

12. MathWorks(R). MATLAB Documentation: Fit Data with a Shallow Neural Network

13. Mündermann, A., C. O. Dyrby, and T. P. Andriacchi. A comparison of measuring mechanical axis alignment using three-dimensional position capture with skin markers and radiographic measurements in patients with bilateral medial compartment knee osteoarthritis. *Knee* 15:480–485, 2008.

14. Nagano, Y., K. Naito, Y. Saho, S. Torii, T. Ogata, K. Nakazawa, M. Akai, and T. Fukubayashi. Association between in vivo knee kinematics during gait and the severity of knee osteoarthritis. *Knee* 19:628–632, 2012.

15. Rajagopal, A., C. L. Dembia, M. S. DeMers, D. D. Delp, J. L. Hicks, and S. L. Delp. Full-Body Musculoskeletal Model for Muscle-Driven Simulation of Human Gait. *IEEE Trans Biomed Eng* 63:2068–2079, 2016.

16. Saxby, D. J., L. Modenese, A. L. Bryant, P. Gerus, B. Killen, K. Fortin, T. v. Wrigley, K. L. Bennell, F. M. Cicuttini, and D. G. Lloyd. Tibiofemoral contact forces during walking, running and sidestepping. *Gait Posture* 49:78–85, 2016.

17. Schreiber, C., and F. Moissenet. A multimodal dataset of human gait at different walking speeds established on injury-free adult participants. *Sci Data* 6:1–7, 2019.

18. Simonsen, E. B., K. L. Cap-Pelen, R. Í. Skorini, T. Alkjaer, K. L. Cappelen, P. K. Larsen, and P. Dyhre-Poulsen. Explanations Pertaining to the Hip Joint Flexor Moment During the Stance Phase of Human Walking. 2012.

19. Taylor, W. R., P. Schütz, G. Bergmann, R. List, B. Postolka, M. Hitz, J. Dymke, P. Damm, G. Duda, H. Gerber, V. Schwachmeyer, S. H. Hosseini Nasab, A. Trepczynski, and I. Kutzner. A comprehensive assessment of the musculoskeletal system: The CAMS-Knee data set. *J Biomech* 65:32–39, 2017.

20. Tohka, J., and M. van Gils. Evaluation of machine learning algorithms for health and wellness applications: A tutorial. *Comput Biol Med* 132:104324, 2021.

21. Vanwanseele, B., D. Parker, and M. Coolican. Frontal knee alignment: Three-dimensional marker positions and clinical assessment. *Clin Orthop Relat Res* 467:504–509, 2009.

22. Winby, C. R., D. G. Lloyd, T. F. Besier, and T. B. Kirk. Muscle and external load contribution to knee joint contact loads during normal gait. *J Biomech* 42:2294–2300, 2009.
